# Evaluation of both overall and individual FMS components results in male and female groups: a systematic review and meta-analysis

**DOI:** 10.3389/fphys.2025.1669967

**Published:** 2026-01-12

**Authors:** Muhammad Ibrar Ahmad, Yi Zhang, Muhammad Talha Younas, Ayesha Parveen, Yu Shi, Yunhang Lu, Zhengxue Song

**Affiliations:** 1 School of Physical Education and Sports Science, Soochow University, Suzhou, China; 2 Department of Physical Education, Kyungpook National University, Daegu, Republic of Korea; 3 Institute of Physical Culture and Sports, Tomsk State University, Tomsk Oblast, Russia; 4 College of Sports Science, Shenyang Normal University, Shenyang, China

**Keywords:** FMS score, functional movement screening, gender difference, individual FMS, male and female

## Abstract

**Objectives:**

This meta-analysis aimed to identify gender-based differences of both overall Functional Movement Screen (FMS) and individual FMS components in male and female groups.

**Methods:**

A comprehensive search was performed across three major databases (PubMed, Web of Science, and Google Scholar) to ensure rigorous inclusion criteria. Data collection took place from 2016 to 2024, and 1,235 articles were identified. After scrutiny, 20 met the requirements for inclusion. The Review Manager 5.4 and CMAv4 software were utilized to examine the FMS score results to ensure rigorous statistical evaluation. Data were synthesized using a random-effect model, with the Mean difference (MD) and 95% confidence interval (CI) used to calculate effect size.

**Results:**

The overall FMS score showed that the functional movement capacities of males and females differed; females performed higher (MD = −0.46, 95% CI = −0.83 to −0.08, P = 0.02) compared to males. A meta-analysis of 7 individual FMS components was conducted to assess the importance for both sexes. However, tests on specific areas showed significant gender differences: females outperformed males in shoulder mobility (p < 0.00001), active straight leg raise (p < 0.00001), hurdle step (p = 0.01), and rotary stability (p = 0.002). In contrast, males demonstrated significantly greater trunk stability (p < 0.0001) compared to females. Despite this, the in-line lunge (p = 0.42) and deep squat (p = 0.20) demonstrated no significant difference across gender.

**Conclusion:**

These outcomes highlight significant gender-based differences that can help identify weaknesses and strengths, which may assist coaches, trainers, and individuals in recommending gender-specific exercises and training programs.

**Systematic Review Registration:**

https://www.crd.york.ac.uk/PROSPERO/display_record.php?RecordID=1043946, identifier CRD420251043946.

## 1 Introduction

Inappropriate posture is commonly associated with various medical conditions, discomfort, and muscular deficiencies, which can result in anatomical changes that ultimately affect the bone structure (Kratenová et al., 2007). As noted by Luijten et al. (2024), athletes with physical impairments often experience high injury rates during training, highlighting the need to identify these deficiencies to prevent long-term complications. Failure to identify and rectify these deficiencies may result in altered movement patterns, reduced performance, and increased injury risk. These movement deficiencies, left unchecked, can exacerbate asymmetries that ultimately lead to inefficient biomechanics and a greater susceptibility to injury.

Several screening methods have been developed to assess movement patterns and identify potential weaknesses before they result in injuries. Early identification of functional deficits was critical for designing personalized training regimens to improve movement quality and reduce injury risk. Initially, these screening tools were primarily sports-specific and often focused on identifying factors that restricted participation certain exercises. They were used to assess sports performance and provide standardized exercise guidelines, which, however, may not align with an athlete’s specific needs. More recently, a functional approach used to develop a personalized training program that enhances or modifies movement patterns based on an individual’s specific needs.

One such approach is the Functional Movement Screen (FMS), developed by Gray Cook and Lee Burton (Gray Cook et al., 2010). The FMS is a biomechanical assessment method that evaluates fundamental movement patterns to identify movement limitations and asymmetries that could impact performance and increase injury risk (Chorba et al., 2010). It includes seven movement tests designed to evaluate fundamental impairments critical for determining physical health and movement quality. Each test assesses specific impairments related to proprioception (body awareness), flexibility, strength, coordination, balance, and mobility (Cook, 2004).

The FMS test assesses a distinct aspect of kinetic efficiency (McMullen and Uhl, 2000). For example, five of the seven tests evaluate each side of the body independently (Cook et al., 2006a; Cook et al., 2006b), making them helpful in detecting asymmetries considered risk factors for injury (Cook et al., 2014a; Cook et al., 2014b). The individual tests included shoulder mobility (SM) assesses muscles, joints, thoracic spine scapula, and range of motion; active straight leg-raise (ASLR), which measures the gastro soleus and flexibility of the hamstrings and their muscles; trunk stability push-up (TSPU), which assesses core stability in the sagittal plane of spine, deep squat (DS) assesses hips, ankles, knees, quadriceps, abductors, and hamstring muscles; hurdle step (HS) assesses the stability as well as mobility of the knee, ankle, and hips muscle; in-line lunge (ILL), which evaluate stability, mobility and symmetry of knee, ankle, feet, and hip muscles; rotary stability (RS) assesses neuromuscular coordination and core stability (Cook et al., 2006a; Cook et al., 2006b; Cook et al., 2014a; Cook et al., 2014b). Each individual test is scored from 0–3: 3 represents perfect movement, 2 indicates movement with some adjustments, 1 indicates difficulty or pain during movement, and 0 signifies the inability to perform the movement. These scores provide an objective assessment of an individual’s physical mobility and stability, offering significant insights about their movement patterns (Cook et al., 2006a; Cook et al., 2006b).

The FMS has been extensively used in rehabilitation and sports performance contexts because of its ability to forecast injury risk and guide training programs (Gulgin and Hoogenboom, 2014). Research has shown a positive correlation between fewer asymmetries and greater flexibility and muscle strength, suggesting that improving these traits could enhance performance and reduce injury risk (Triplett et al., 2021). Nevertheless, despite the tool’s extensive use, there remains a lack of consensus in the literature addressing the existence and magnitude of gender-based disparities in FMS scores (Chimera et al., 2017; Chimera et al., 2015). Some studies, such as those by (Domaradzki and Koźlenia, 2020) found that males performed better in core stability and strength, whereas other studies suggested that females might achieve higher overall scores (Gavigan et al., 2024; Thomas et al., 2022). However, some studies have failed to confirm significant gender differences (Perry and Koehle, 2013; Schneiders et al., 2011), highlighting the contradictory nature of the existing evidence. Furthermore, previous meta-analyses have been performed to assess the reliability (Bhudarally et al., 2025) of FMS as a predictive tool for injury risk, while some of them support its validity (Bonazza et al., 2017), others report non-significant results in using FMS for injury prediction (Dorrel et al., 2015). While only one study by O'Brien et al. was among the first to conduct a meta-analysis examining gender variations in FMS scores. However, their findings showed inconsistencies in gender differences observed across subgroups (O'Brien et al., 2022).

Therefore, this meta-analysis attempts to identify these discrepancies by examining gender-based differences in overall and individual FMS components scores. Specifically, it aims to determine which individual FMS assessments exhibit significant gender differences, thereby contributing to a deeper understanding of how gender affects individual functional movement patterns and guiding training and injury-prevention techniques. By addressing these gaps, the findings from this meta-analysis may contribute to developing more specific interventions and screening techniques, that enhance movement quality and optimize athletic performance; to understanding how gender-specific movement patterns lead to better, customized training and injury-reduction programs; and to optimizing more efficient movement patterns for female and male athletes.

## 2 Methods

**FIGURE 1:**
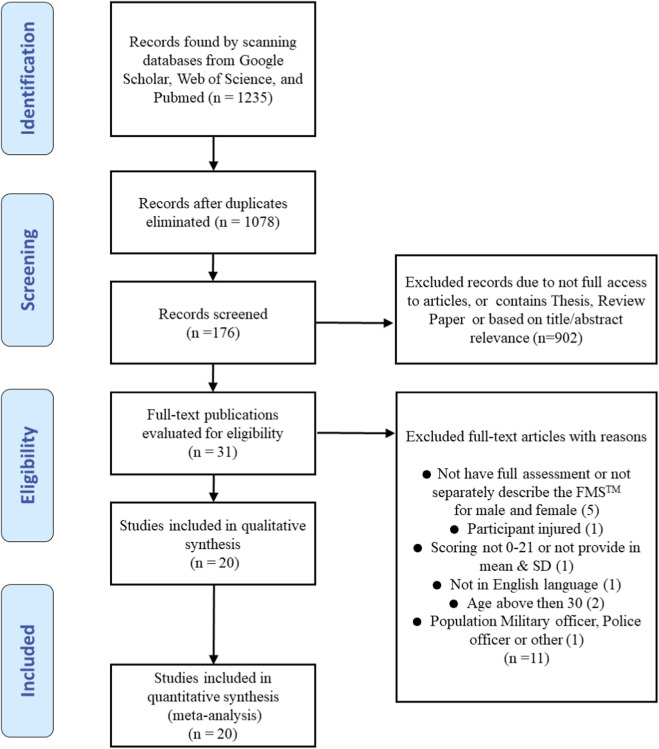
Flow diagram of included studies.

A systematic literature search was conducted following the Preferred Reporting Items for Systematic Reviews and Meta-Analyses (PRISMA) recommendations to ensure a rigorous and transparent methodological approach (Figure 1); (Liberati et al., 2009). The analysis has been registered with PROSPERO, CRD420251043946.

### 2.1 Search strategy

To comprehensively capture relevant studies, an exhaustive search was executed across three databases: PubMed, Web of Science, and Google Scholar, focusing on evaluating FMS scores in both male and female participants. The search was confined to studies published between 2016 and 2024 to ensure the inclusion of contemporary studies. The study concentrated on FMS scores, specifically examining 7 individual FMS test scores for both genders. The search query incorporated terms such as (FMS score* OR functional movement screening* OR quality movement* OR movement pattern* OR FMS individual test) AND (individual FMS OR functional ability) AND (school students* OR college students*) AND (gender difference* OR sex difference*), AND (male* OR female*), see Supplementary Table 1. In PubMed, the search strategy employed title/abstract.

Two independent researchers conducted the literature search and screening process to ensure comprehensive retrieval. Any discrepancies in article selection were resolved through consensus discussions. In addition, manual searches and reference list screening were also performed to reduce the probability of overlooking pertinent articles. These electronic and manual searches ensured a comprehensive coverage of relevant studies.

### 2.2 Eligibility criteria

The eligibility criteria for this analysis follow the PRISMA guidelines and the PICO model was used to evaluate inclusion criteria. Population: The studies are considered to be included if they report: (1) Population: male and female participants aged 6–30 years, representing school-aged students, university students, and athletes. Participant must remain uninjured for at least 1 month before performing the Functional Movement Screen tests; (2) Intervention/exposure: The exposure variable was biological sex (male vs. female); (3) Outcomes: The primary outcomes for eligibility criteria were: studies must report the overall FMS test score results, using standard 0–21 scoring scale; Secondary outcomes: include the seven sub-tests (SM, ASLR, TSPU, DS, HS, ILL, RS); (4) Comparator: opposite gender (male vs. female); (5) Study design: peer-reviewed full text observational studies, such as cross-sectional studies, cohort studies, and retrospective studies; (6) Included studies must be in English between 2016 and 2024. Exclusion Criteria: (1) Non-peer-reviewed publication; (2) Books, review papers, abstracts, conference proceedings, and theses or dissertations; (3) firefighters, police officers, army officers, and other similar groups; (4) inaccessibility to relevant information. Study characteristics contained the publishing year, country of study, sample size, age range, statistical methods, and participant category.

### 2.3 Data extraction and screening

The research articles’ findings were meticulously extracted to ensure the accurate collection of relevant data from each study. Key study characteristics, including study year, country of conduct, sample size, age range, statistical techniques, and participant category, were meticulously documented. Two researchers independently extracted data, including the mean and standard deviation (SD) of FMS test results, and the scores of both genders on the seven FMS test components. To collect data, the full-text papers of potentially relevant research were gathered and meticulously evaluated according to the pre-defined standards for inclusion. During the screening phase of this meta-analysis, two researchers (MIA and YZ) independently scrutinized the titles and abstracts of publications for relevance to the review. All extracted data were cross-checked independently, and discrepancies were resolved by the two authors. If the consensus was not resolved, a third reviewer (LY) was consulted for assistance. In studies with missing key information, mixed-gender samples, or incomplete gender-specific data, we attempted to contact the original authors for additional data; if unavailable, studies were evaluated for possible exclusion according to established criteria. This comprehensive screening approach ensured that only high-quality, relevant publications were included in this meta-analysis.

### 2.4 Subgroup analysis

A subgroup analysis was performed to evaluate whether age, sports participation, and region could impact the overall FMS scores. Age was categorized into two groups: younger (6–17 years) and adult (17–30 years). Sports participation was categorized into sports participation and non-sporting active participant groups to analyze whether participation in specific sports could affect the FMS scores. The region was categorized into three groups: Europe, North America, and Asia, to evaluate whether different environments, cultures, and physical activity infrastructures could impact FMS scores of both genders.

### 2.5 Standards for evaluating risk of bias

To determine the potential for bias in the identified studies, the Newcastle-Ottawa Scale (Peterson et al., 2011) was used, focusing on non-randomized bias criteria given the predominantly cross-sectional nature of our meta-analysis (Herzog et al., 2013). The risk of bias was assessed across three domains: (1) selection: included 3 criteria; representativeness of the sample, sample size justification, non-respondents, ascertain of exposure, (2) comparability: control for confounding, and (3) outcomes: Included three criteria: assessment method of FMS tests, blinding of outcome assessors, statistical test appropriateness. Regarding these principles, the authors assessed each study and rated each NOS criterion (except control of confounding) as “1” (indicating the criterion was met) or “0” (indicating the criterion was not met). The control for confounding and ascertain of exposure criterion was rated as “2” (indicates the requirement for criterion met), “1” (indicates the requirement for criterion partially met), and “0” for not met. This rigorous evaluation facilitated a comprehensive evaluation of the risk of bias in this analysis. Each domain contains a set of star scores that facilitate the evaluation of bias risk linked to this standard. The threshold for scoring the overall quality of studies was as follows: Low quality, a score below 5; Moderate quality, a score of 5–6; High quality, a score of 7–10. This systematic strategy offered a strong framework for assessing potential bias sources and ensured that the meta-analysis results originated from research of adequate rigor.

### 2.6 Statistical analysis

A total of 19 studies met the inclusion criteria, focusing on the analysis of the overall FMS test, while 12 studies analyzed individual FMS components, aiming to compare these scores across male and female groups to investigate potential gender differences. The Review Manager 5.4 (Cochrane Collaboration, Copenhagen, Denmark) software was used for statistical analysis, to ensure statistical rigor and reliability (Manager, 2014). The Mean difference (MD) and confidence intervals 95% CI were used as effect size for all continuous outcome measures, as all included studies reported same the 0–21 scale for measuring FMS scores. A positive MD indicates that males performed better in the FMS scores compared to females. While negative MD indicates females performed higher than males. The 95% CI indicates the precision of the pooled estimate. We also calculated 95% prediction intervals (PI) to assess the potential heterogeneity of effect size across different studies. The subgroup analysis was conducted to explore the source of variance.

The data were analyzed using a random-effects framework with the DerSimonian-Laird adjustment to account for expected heterogeneity (tau^2^) across studies, considering differences in age, sample characteristics, physical activity level, and region (Borenstein et al., 2010; DerSimonian and Laird, 1986). These factors contribute to the heterogeneity across studies, and a random effect model was chosen because it accommodates the heterogeneity (Borenstein et al., 2010). The DerSimonian and Laird technique does not presume any certain distribution for the random effects. This adjustment has proven to be an effective method for handling heterogeneity across studies, especially when significant diversity is present.

Heterogeneity among studies was assessed using the tau^2^ statistic, Chi^2^ test, and the I^2^ statistic (Higgins, 2008). A high value of tau^2^ indicates greater heterogeneity across studies. Statistical significance heterogeneity was measured using the Chi^2^ test with a significance threshold set at p < 0.10 due to the test’s low statistical power when studies are few or have small sample sizes. The I^2^ statistic was used to evaluate the extent of the heterogeneity, with the following interpretation: values between 0% and 30% indicate no heterogeneity, values between 30% and 50% indicate moderate heterogeneity, and values between 50% and 75% indicate substantial heterogeneity, and values exceeding 75% indicate high heterogeneity (Chandler et al., 2019; Cumpston et al., 2019).

For further robustness, Comprehensive Meta-Analysis (CMA) version 4 (Biostat, Englewood, NJ) software was used to perform Egger’s test for the detection of publication bias, and trim-and-fill analysis was used to adjust for missing studies and evaluate the possible influence on effect size estimates (Borenstein, 2022). Finally, a sensitivity analysis was conducted by omitting each included study to verify the reliability of the findings. This approach guarantees the robustness and generalizability of the results by assessing whether any single study unduly affected the aggregated outcomes.

## 3 Results

### 3.1 Literature selection

Initially, 1,235 relevant articles were identified using the search strategy, of which 157 were duplicates, leaving 1,078 for further examination. After screening, 176 records were assessed, of which 902 were excluded due to lack of full article access, being theses or review papers, or for title/abstract relevance. Of the 176 records, 31 full-text publications were evaluated for eligibility to be included in the study. An additional 11 articles were excluded after a detailed assessment, with reasons: incomplete FMS Composite Score assessment, no separate description of FMS scores for males and females, use of a scoring system different from 0–21, not being in English, participants being over 30 years old, or the study population consisting of military or police officers or other specific groups. Ultimately, 20 papers met the requirements for inclusion (Boguszewski et al., 2017; Domaradzki and Koźlenia, 2020; García-Luna et al., 2020; Garcia-Pinillos et al., 2019; Gnacinski et al., 2016; Karuc et al., 2020; Kramer et al., 2019; Lim et al., 2020; Martín-Moya et al., 2023; Pachava and Bisht, 2024; Pfeifer et al., 2019; Philpott et al., 2023; Razi, 2016; Samer et al., 2024; Triplett et al., 2018; Vehrs et al., 2021; Vernetta-Santana et al., 2019; Vujkov et al., 2024; Wright and Chesterton, 2019; Wu et al., 2021). Of these, 19 studies provided data for the FMS overall test, while one study provided data only for individual FMS components; thus, it was excluded from the overall FMS score. In total, twelve studies fulfilled the inclusion criteria for the individual FMS components analysis (Figure 1).

### 3.2 Study characteristics

This review compiled 20 studies published between 2016 and 2024. The author did a verification scan to extract accurate demographic and descriptive data. Participants’ ages ranged from school-going children (6–30 years) to university students. The research encompassed several countries that represent diverse populations. The studies included participants predominantly from school-aged groups, with particular age ranges and educational levels outlined as follows:

Out of 20 studies three were conducted in US (Gnacinski et al., 2016; Kramer et al., 2019; Triplett et al., 2018) and Spain (Garcia-Pinillos et al., 2019; Martín-Moya et al., 2023; Vernetta-Santana et al., 2019), two in UK (Pfeifer et al., 2019; Wright and Chesterton, 2019) and in Poland (Boguszewski et al., 2017; Domaradzki and Koźlenia, 2020), and one in Iran (Razi, 2016), Ireland (Philpott et al., 2023), India (Pachava and Bisht, 2024), Croatia (Karuc et al., 2020), Korea (Lim et al., 2020), Germany (Vehrs et al., 2021), Serbia (Vujkov et al., 2024), Palestine (Samer et al., 2024), China (Wu et al., 2021), and in Paris (García-Luna et al., 2020). This review includes research conducted on school students, university and college students, soccer players, hockey players, and Taekwondo athletes. Specifically, 12 studies have an age range from 6 to 18, while the others have an age range from 18 to 30. Regarding the sample size, the studies included participants ranging from 28 to over 700. Currently, two studies recruited large sample, while four recruited samples below 40. In the research by (Boguszewski et al., 2017), the total number of participants was 104, but in the present analysis, we considered only hockey players’ FMS outcomes. This review shows several populations and methodologies for evaluating FMS across settings and age groups. The studies came from various countries, which represent a diverse set. The studies included participants predominantly from school-aged groups, with particular age ranges and educational levels outlined in (Table 1).

**TABLE 1:** Descriptive characteristics of included studies.

Study	Year	Age	Country	N	Male, female	Study design	Statistical techniques	Population characteristics
Boguszewski et al. (2017)	2017	10–15 years	Poland	104	88, 16	CS	MW U	Hockey players and non-hockey players
Triplett et al. (2018)	2018	18–26 years	US	100	43, 57	RS	PCA	university students
Domaradzki and Koźlenia (2020)	2020	19–25 years	Poland	89	42, 47	CS	MW U	University school of physical education
Garcia-Pinillos et al. (2019)	2019	6–11 years	Spain	172	89, 83	CS	ANCOVA, and MLR	School Child in southern Spain
García-Luna et al. (2020)	2020	Under 13–16	Paris	30	14, 16	CS	IND-T	Chidaoba Judo Club/Athletes’ School
Gnacinski et al. (2016)	2016	18–23 years	US	176	88, 88	CS	IND-T	College athletes
Karuc et al. (2020)	2020	16–17 years	Croatia	725	359, 366	CS	MLM	Public, private and vocational school adolescents
Kramer et al. (2019)	2019	16–17 years	US	56	28, 28	ChS	IND-T, PCA	High school athletes
Lim et al. (2020)	2020	14–15 years	Korea	38	19, 19	CS	IND-T	Soccer players
Martín-Moya et al. (2023)	2023	19–23 years	Spain	28	14, 14	CS	IND-T, MR	semi-professional soccer players
Pachava and Bisht (2024)	2024	18–30 years	India	50	32, 18	CS	IND-T T-Test	University athletes
Pfeifer et al. (2019)	2019	11–18 years	UK	136	63, 73	ChS	IND-T	Local high school and sports organization
Philpott et al. (2023)	2023	12–16 years	Ireland	364	131, 129	CS	*t*-tests, ANCOVA	six Irish secondary schools
Razi (2016)	2016	19–27 years	Iran	45	24, 21	CS	IND-T, Chi-square	healthy taekwondo athletes
Samer et al. (2024)	2024	20–25 years	Palestine	139	66, 73	CS	IND-T	Sports sciences and physical education students
Vehrs et al. (2021)	2021	10–12 years	Germany	94	53, 41	CS	ANCOVA	School Children involve in PE
Vernetta-Santana et al. (2019)	2019	12–13 years	Spain	35	11, 24	CS	MW U	Secondary school adolescents
Vujkov et al. (2024)	2024	21–25 years	Serbia	99	49, 50	CS	MRA	College students
Wright and Chesterton (2019)	2019	8–18 years	UK	144	48, 96	CS	GL, PRM	Young athletes sports and track and field athletics
Wu et al. (2021)	2021	7–10 years	China	117	78, 39	CS	MW U	Primary school children

CS, Cross-sectional; ChS, cohort study; RS, Retrospective study; MW U, mann whitney u test; PCA, pearson correlation analysis; MLR, multiple linear regression, independent t-test; MLM, multilevel modeling; MRA, multivariate regression analysis; GL, general linear; PRM, pearson correlation matrix; NOS, Newcastle-Ottawa Scale.

### 3.3 Risk of bias

The NOS bias assessment in the included studies, comprising 20 studies, indicates that all reported a low risk of bias, with scores ranging from 7 to 9. In the selection bias domain, 2 studies received 5 stars, 10 received 4 stars, and 8 received 3 stars. Specifically, all studies reported on sample representativeness, handling of non-respondents, assessment of exposure, and sample justification, with 7 studies scoring 0 in the non-respondent category, and 9 in the sample justification category. To address comparability bias, all studies reported a low risk, indicating that they controlled for potential confounding factors appropriately. In the outcome bias domain, all studies assessed two aspects related to outcome evaluation. Specifically, all studies thoroughly evaluated the assessment of outcomes and the appropriateness of statistical test appropriateness thoroughly, indicating a consistent and reliable approach to analyzing the data (Table 2).

**TABLE 2:** Risk of bias table.

Study	Selection bias	Comparability	Outcome bias	Risk level
Boguszewski et al. (2017)	****	**	**	8
Triplett et al. (2018)	****	**	**	8
Domaradzki and Koźlenia (2020)	****	**	**	8
Garcia-Pinillos et al. (2019)	***	**	**	7
García-Luna et al. (2020)	***	**	**	7
Gnacinski et al. (2016)	***	**	**	7
Karuc et al. (2020)	*****	**	**	9
Kramer et al. (2019)	***	**	**	8
Lim et al. (2020)	***	**	**	7
Martín-Moya et al. (2023)	****	**	**	8
Pachava and Bisht (2024)	****	**	**	8
Pfeifer et al. (2019)	****	**	**	8
Philpott et al. (2023)	****	**	**	8
Razi (2016)	****	**	**	8
Samer et al. (2024)	****	**	**	8
Vehrs et al. (2021)	***	**	**	7
Vernetta-Santana et al. (2019)	***	**	**	7
Vujkov et al. (2024)	***	**	**	7
Wright and Chesterton (2019)	****	**	**	8
Wu et al. (2021)	*****	**	**	9

### 3.4 Meta-analysis

#### 3.4.1 Overall FMS score between genders

**FIGURE 2:**
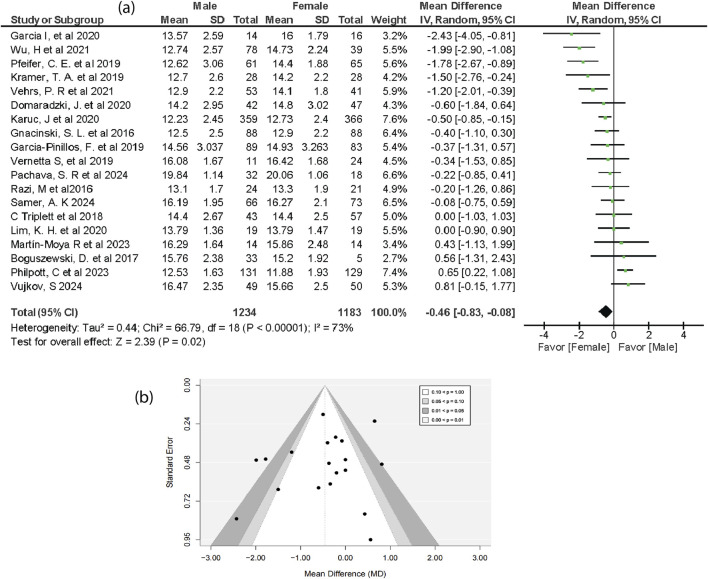
Forest plot **(a)** and Funnel plot **(b)** of FMS mean in male and female groups.

Both male and female groups showed substantial mean differences in functional movement screen results across 19 studies, with males n = 1,234 and females n = 1,183. More specifically, in this meta-analysis both sexes demonstrated a notable mean difference. The pooled results showed that females performed significantly better than males, with an overall mean difference of (MD = −0.46, 95% CI = −0.83 to −0.08, 95% PI = −1.92 to 1.00, P = 0.02) (Figure 2). The studies revealed substantial heterogeneity (I^2^ = 73%), suggesting variability in the effect sizes across different populations. Although the small-to-moderate effect size of −0.46 was not sufficient to clearly define individuals’ fundamental abilities, recognizing this small but meaningful difference can help coaches, trainers, and clinicians tailor group-based intervention programs that better address the specific needs of both genders. The 95% prediction interval suggests that the effect in future similar studies lies between −1.92 and 1.00, which is wider than the confidence interval.

The vertical line in the forest plot represents the no-effect line, and the diamond at the bottom reflects the pooled overall effect size across the included studies. In the funnel plot, Most studies cluster around the mean effect size at the top of the graph. Smaller studies with higher sampling variance in effect size estimates are shown at the bottom of the graph and are scattered throughout a range of values. However, the funnel plot showed no evidence of publication bias (Figure 2). Egger’s regression test supported the absence of publication bias (p = 0.135). Additionally, the trim-and-fill analysis showed no significant change in effect size. The figures for the sensitivity analysis and Egger’s regression for the overall FMS and Individual FMS test results are provided in (Supplementary Table 2).

#### 3.4.2 Subgroup analysis FMS score between genders

The subgroup analysis was conducted to further investigate the interpretations of the overall FMS score and to highlight the source of heterogeneity.

Age was analyzed to examine the impact of younger (6–17 years) and adult (18–30 years) age groups on overall Functional Movement Screen (FMS) scores. This subgroup analysis showed that younger individuals (6–17 years) exhibited a statistically significant difference in favor of females (p = 0.01) (Table 3), whereas in adults, there was no statistically significant difference in overall FMS score between genders (p = 0.53) (Table 3). A high degree of heterogeneity, I^2^ = 83% was observed in the younger (6–17 years) group; in contrast, no heterogeneity, I^2^ = 0% was observed in the adult (18–30 years) group, while the total subgroup difference showed high heterogeneity, I^2^ = 83% (Supplementary Table 2).

**TABLE 3:** Subgroup analysis.

Subgroup analysis	Subgroup category	Sample sizes (M and F)	Weights	Mean difference (MD)	95% CI		p value	I^2^
					Lower	Upper		
Age	Younger (6–17 years)	876,815	57.50%	−0.78	−1.37	−0.18	0.01	83%
	Adults (18–30 years)	358,368	42.50%	−0.1	−0.4	0.21	0.53	0%
Sports participation	Sports participants	738,713	57.00%	−0.52	−0.91	−0.13	0.009	55%
	Non-sporting AP	540,457	43.00%	−0.44	−1.2	−0.32	0.26	83%
Region-based	Europe	856,840	55.20%	−0.42	−0.99	0.15	0.15	79%
	North America	159,173	15.70%	−0.53	−1.26	0.19	0.15	41%
	Asia	219,170	29.10%	−0.48	−1.15	0.2	0.17	71%

M, male; F, female; CI, Confidence interval; AP, active participants.

In subgroup analysis, sports participants showed a significant difference (p = 0.009) in overall FMS scores (Table 3), with a female advantage. Whereas among non-sporting active participants, no significant difference (p = 0.26) was observed across both genders. Sports participants showed substantial heterogeneity I^2^ = 55%, whereas non-sporting active participants exhibited high heterogeneity I^2^ = 83%. Overall, the total subgroup difference showed no heterogeneity I^2^ = 0% (Supplementary Table 2).

In the region-based subgroup analysis, no significant difference in overall FMS scores was found between both genders (p > 0.05) (Table 3). No heterogeneity (I^2^ = 0%) was observed in the total subgroup difference (Supplementary Table 2).

#### 3.4.3 Individual FMS components score between genders

For the individual FMS, 12 studies containing data for both sexes were meta-analyzed. The analysis focuses on identifying specific tests that exhibit noteworthy gender differences.

##### 3.4.3.1 Shoulder mobility

**FIGURE 3:**
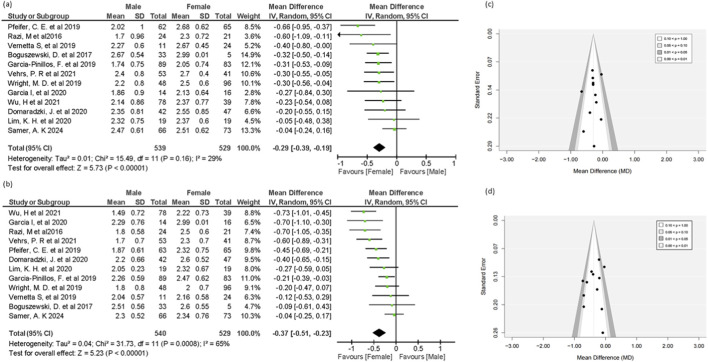
Forest plot of **(a)** shoulder mobility test and **(b)** active straight-leg raise test mean in male and female groups, funnel plot of **(c)** shoulder mobility test and **(d)** active straight-leg raise test.

Twelve out of twenty studies reported relevant data included in this analysis. The results suggest a notable difference, with females demonstrating a significant advantage over males (MD = -0.29, 95% CI = -0.39 to -0.19, 95% PI = -0.54 to -0.04, p < 0.00001) (Figure 3). A small-to-moderate effect size of -0.29 indicated a slight but significant finding. The result showed the greater flexibility and joint laxity in females, as required during the shoulder mobility test. The result showed no heterogeneity (I^2^ = 29%). The funnel plot showed no obvious asymmetry, indicating no publication bias. Further, Egger’s test confirmed the absence of publication bias (p = 0.414).

##### 3.4.3.2 Active straight leg raise

Twelve out of twenty studies reported relevant data included in this analysis*.* A statistically significant advantage for females was found in the (ASLR) test scores comparing males (MD = −0.37, 95% CI = −0.51 to −0.23, *95% PI = -0.84 to 0.10,* p < 0.00001) (Figure 3). A moderate effect size of −0.37 indicated that females are better at stretching the hamstrings, gastrocnemius, and soleus, as required during the ASLR test. Substantial heterogeneity (I^2^ = 69%) was observed. The 95% prediction interval (−0.84 to 0.10) showed a wider true effect. The funnel plot appeared largely symmetrical. Egger’s test showed no publication bias (p = 0.359).

##### 3.4.3.3 Hurdle step test

**FIGURE 4:**
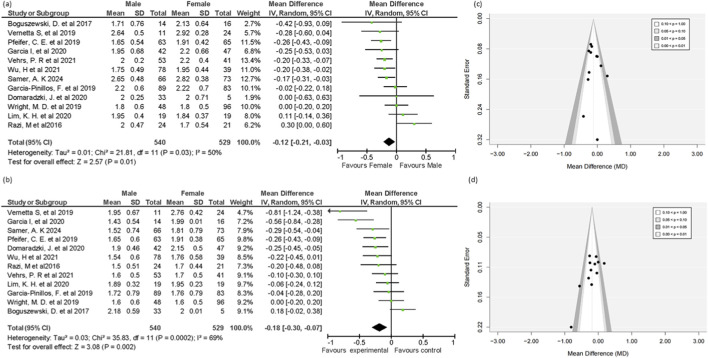
Forest plot of **(a)** Hurdle step test and **(b)** Rotary stability test mean in male and female groups, funnel plot of **(c)** Hurdle step test and **(d)** Rotary stability test.

Twelve out of twenty studies reported relevant data included in this analysis. The outcomes of the hurdle step test indicate results favoring the female group over males (MD = −0.12, 95% CI = −0.21 to −0.03, *95% PI = -0.37 to 0.13,* p = 0.01), with moderate heterogeneity (I^2^ = 50%) (Figure 4). The small but meaningful effect size of −0.12 indicated that females showed greater stability and mobility in knee, ankle, and hip joints. Funnel plot appeared largely symmetrical, which indicates no evidence of publication bias, and egger’s test further supports this (p = 0.394).

##### 3.4.3.4 Rotary stability

In the rotary stability analysis, a substantial difference was found. Twelve of the twenty studies report data for a forest plot in this meta-analysis. The forest plot of this analysis showed a significant mean difference (MD = −0.18, 95% CI = −0.30 to −0.07, *95% PI = -0.59 to 0.23,* p = 0.002), suggesting that females exhibited greater neuromuscular coordination during this FMS test than males (Figure 4). The 95% PI showed a wider effect compared to 95% CI. Substantial heterogeneity (I^2^ = 69%) was observed. The funnel plot showed symmetrical distribution, indicating no significant bias, and Egger’s test further supports this (p = 0.316).

##### 3.4.3.5 Trunk stability test

**FIGURE 5:**
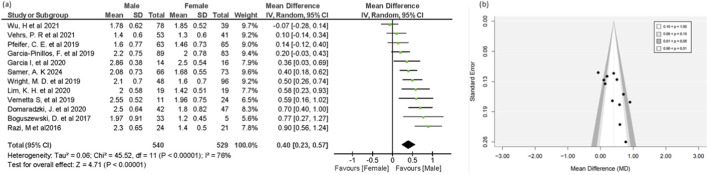
**(a)** Forest plot and **(b)** funnel plot of Trunk Stability test mean difference in male and female groups.

Twelve out of twenty studies reported sufficient data to be included in this analysis. The pooled mean difference for the trunk stability (TSPU) test (MD = 0.40, 95% CI = 0.23 to 0.57, 95% PI = −0.18 to 0.98, p < 0.00001), indicating males had a higher performance level in the TSPU test compared to females (Figure 5). This moderate effect size of 0.40 suggests that, in terms of trunk stability, men typically perform greater than women. Due to the substantial heterogeneity (73%), the funnel plot was created. The funnel plot appeared largely symmetrical, suggesting no significant evidence of bias in the studies used in this analysis. However, Egger’s test showed a marginally significant p-value of 0.082, indicating the possibility of slight publication bias. Nonetheless, as the p-value was greater than 0.05, meaning any bias, if present, was minimal.

Under the random-effect model, the trim-and-fill analysis produced a point estimate, and the 95% confidence interval for the combined studies is 0.38926 (0.22384, 0.55468). After adjusting for missing studies, the imputed point estimate decreased to 0.29086 (0.11958, 0.46214). This reduction from 0.38926 to 0.29086 suggests that publication bias due to missing studies may have exaggerated the original effect size. The findings after imputation are more conservative and indicate a somewhat weaker correlation (Supplementary File 2).

#### 3.4.4 Non-significant test (DS and ILL)

**FIGURE 6:**
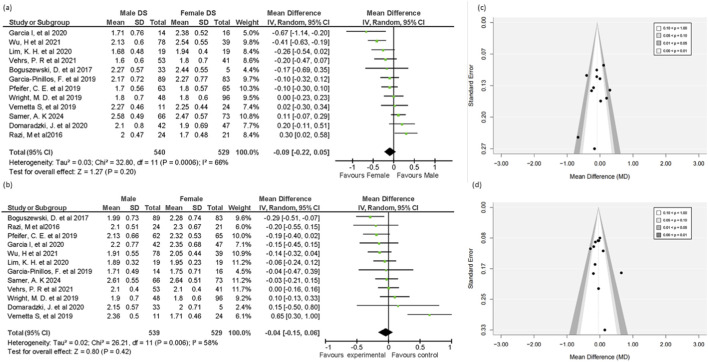
Forest plot of **(a)** deep squat test **(b)** in-line lunge test mean difference in male and female groups, Funnel plot of **(c)** deep squat and **(d)** in-line lunge test.

According to the findings from twelve studies that reported sufficient data, there are no gender-specific statistically significant variations in the DS and ILL tests (MD = −0.09, 95% CI = −0.22 to 0.05, 95% PI = −0.51 to 0.33, p = 0.20), (MD = −0.04, 95% CI = −0.15 to 0.06, 95% PI = −0.37 to 0.29, p = 0.42), respectively (Figure 6). A substantial heterogeneity was found in both DS and ILL tests (I^2^ = 66%, I^2^ = 58%), respectively (Figure 6). Although two studies (García-Luna et al., 2020; Wu et al., 2021) out of 12 included in this test reported higher performance from females in the deep squat test compared to males, while one study (Razi, 2016) reported in favor of males, these differences were insufficient to reach statistical significance. The diamond shape in the forest plot, representing the overall effect size, slightly leans to the left, indicating a small effect in favor of females; however, this effect was not significant (p = 0.20). Funnel plot showed symmetrical representation, which indicates no significant bias, and Egger’s test further supports this (p = 0.453).

Similarly, for the ILL test, one study reported results favoring females (Boguszewski et al., 2017), while another study found better performance in males (Vernetta-Santana et al., 2019). However, neither of these differences was statistically significant (p = 0 0.42). These suggest that neither the DS nor the ILL demonstrated a significant gender-based performance difference. These results are likely due to the test design and the traits of the individuals involved in the studies (Figure 6). The Funnel plot appeared largely symmetrical, which indicates no significant bias, and Egger’s test further supports this (p = 0.474).

## 4 Discussion

This meta-analysis sought to determine how males and females of school and university-aged populations differed in their FMS composite scores. Systematically reviewing and quantitatively synthesizing published FMS data from male and female cohorts, this study shows gender differences in FMS scores, specifically in individual FMS tests: four out of seven tests (SM, ASLR, HS, and RS) showed results favoring females. Conversely, only one test (TSPU) demonstrated significant male-specific outcomes, while two tests (DS and ILL) exhibited no notable gender-based difference (Table 4).

**TABLE 4:** Individual FMS components score between both genders.

FMS components	Sample sizes (M &F)	Mean difference (MD)	95% CI		p value	I^2^
			Lower	Upper		
SM	539,529	−0.29	−0.39	−0.19	p < 0.00001	29%
ASLR	540,529	−0.37	−0.51	−0.23	p = 0.0008	65%
HS	540,529	−0.12	−0.21	−0.03	p = 0.03	50%
RS	540,529	−0.18	−0.31	−0.07	p = 0.0002	69%
TSPU	540,529	0.4	0.23	0.57	p < 0.00001	76%
DS	540,529	−0.09	−0.22	−0.05	p = 0.20	66%
ILL	539,529	−0.04	−0.15	−0.06	p = 0.42	58%

SM, shoulder mobility; ASLR, Active straight leg-raise; HS, hurdle step; RS, rotary stability; TSPU, Trunk stability push-up; DS, deep squat; ILL, In-line lunge.

### 4.1 Difference in overall FMS score between genders

The establishment of a composite score of FMS test results between genders yields important insights in this systematic review and meta-analysis. This comprehensive analysis covers a vast range of population data across continents, including Europe, Asia, and North America. Additionally, it incorporates individuals from various professional backgrounds, such as school children, athletes, and university students. By incorporating such diverse data, improve the robustness of the study, enabling universal, comprehensive results for the FMS score across both genders. While (Maloney, 2019) reviews opposes the notion that bilateral asymmetries may not impair performance and suggest that natural asymmetries could be embraced in training, it is generally acknowledged that excessive asymmetries could increase injury risk. Movement asymmetries can also be reduced by participating in various sports (Triplett et al., 2021). For instance, some studies report that asymmetrical movement performance increased injury risk by 2.3 times, particularly when lower FMS scores were observed (Kiesel et al., 2007). was among the first to identify the correlation between low FMS score and an injury risk, especially in a professional football player. Despite several meta-analyses confirming the reliability of FMS as an injury predictive tool (Bonazza et al., 2017), variations in the FMS cut-off score (Chorba et al., 2010; Zarei et al., 2022) can lead to misinterpretations. Based on the findings of this analysis, it appears that males may be at greater injury risk than females. This may also lead to the increased prevalence of poor posture in men, a symptom more often seen in boys (Kratenová et al., 2007).

The differences in FMS scores observed in females may be attributed to factors, including superior joint flexibility (Yu et al., 2022), which results in improved performance on the FMS test. Greater flexibility may be attributed to joint laxity and hormonal influences. Specifically, the function of estrogen is to enhance joint laxity, which is more prevalent in females than in males (Silke, 2011). While joint laxity can improve flexibility and movement control (Bhudarally et al., 2025), one should note that the advantages of increased flexibility are most clearly shown when balanced with strength training. Strength training can improve stability and prevent injury. Therefore, there is a need to emphasize the importance of strength training to negate these possible drawbacks.

Furthermore, the study indicated that repetitive loading during exercise might enhance joint laxity, enabling females to achieve improved movement control and performance (Silke, 2011), strengthening their dominance in these areas compared to males. The relationship between flexibility and strength training is essential for maximizing the enhanced flexibility seen in females. These findings support the notion that females are better at performing symmetrical movement patterns. Therefore, the results of this meta-analysis suggest that females exhibit superior movement patterns in specific domains compared to males, emphasizing the importance of tailored training that addresses gender-specific strengths and weaknesses.

However, individuals with a cut-off score of ≤14 on the FMS are considered to have deficient movement competence attributable to restricted joint mobility, poor joint stability, and movement control. Research suggests that these movement deficiencies can be minimized by using different modes of resistance training (Yildiz et al., 2019). Moreover, one study reports that 8 and 12 weeks of training program significantly improve range of motion, stability, and movement control, leading to improved FMS (Jafari et al., 2020). Several studies have explored the relationship between exercise and the FMS scores. A study (Lim et al., 2024) found that FMS score significantly improved after 8 weeks of yoga and Pilates, compared to before exercise performance. A recent meta-analysis on exercise and FMS scores revealed that resistance training, integrated training, the FIFA11+ program (an injury-prevention program for soccer players), neuromuscular training, and core stability training all significantly improved FMS scores (Maleki et al., 2025). Moreover, the impact of functional strength training (FST) on students’ FMS performance was assessed. The study showed that 12 weeks of FST (including squat, push-up, get up, hip bridge, lunge, hip hinge, upper body pull, windmill) in the experimental group improved overall FMS scores compared with the control group. These findings demonstrate the significant impact of exercise on FMS score. This may help coaches and trainers specify customized training and exercise programs to improve movement asymmetries in both genders. Females might benefit from strength-based training programs to improve FMS tasks that require strength, while males could benefit from mobility and flexibility-based training. These customized approaches would help reduce movement asymmetries, improve functional movement, mitigate injury risk, and close the gender gap in movement performance.

Furthermore, Gender disparities in movement patterns could also be influenced by anthropometric measurements and age-related variables. Numerous studies included in this analysis were conducted on a population aged 7–18, during which biological maturation influences factors such as physical performance, muscle mass, and intramuscular coordination. Studies have reported that females are more mature in this age range compared to males, which may affect our results. Moreover, the forest plot reveals substantial heterogeneity (I^2^ = 73%) between the studies, suggesting that sample size, body mass index BMI, and study environment may have impacted the results. This Heterogeneity was addressed by conducting a sensitivity analysis. After excluding the studies by (García-Luna et al., 2020; Philpott et al., 2023; Wu et al., 2021), the heterogeneity decreased below 50%. The (Philpott et al., 2023) study evaluates FMS between different school grades, which may explain some of the heterogeneity across studies. In this meta-analysis, we combined all data, which could contribute to heterogeneity, but excluding grade-specific data by genders might lead to misinterpretation or incomplete interpretations. The study by (Wu et al., 2021), explored the relationship between motor fitness and the FMS test, which could result in significant differences in movement abilities due to proficiency in movement skills. Additionally, this study had an imbalanced distribution of participants (78 males and 39 females), which could also contribute to the heterogeneity in this analysis. The study (García-Luna et al., 2020) focused on judo athletes, a different population, and had a small sample size (14 males and 16 females) compared to the other studies incorporated in this analysis. The specific training and movement patterns of judo athletes, among other characteristics, may have further increased heterogeneity.

The total sample size in this meta-analysis was 2,417, including 1,234 males and 1,183 females. Notably, two studies had large sample sizes of 725 and 364, which could have influenced the overall results (Karuc et al., 2020; Philpott et al., 2023). Most studies have a small sample size, ranging from 50 to 175 participants, with some having fewer than 50. Study participant background also plays a critical role in results, as some studies focused on the general student population, while others included athletes or professional players. This variation in participant background, particularly in physical activity, could have introduced bias, as students with low physical activity involvement may not yield representative outcomes. Therefore, further research is needed to determine whether these gender differences persist in older populations, where age-related factors and anthropometric changes may play a significant role.

Subgroup analyses by age, sport participation, and region were also conducted to further examine differences in FMS scores. Age subgroup showed the significant gender difference in the younger (6–17 years), while no significant gender differences were observed in the adults (18–30 years) group, suggesting that younger females are better performers in FMS scores. The lack of heterogeneity in the adult group suggests that studies of younger participants may show heterogeneity across studies. Studies suggested that age was a key factor in the FMS test.

In the sports participation subgroup, participants performed significantly better than non-sporting active participants. This suggests that individuals who engage in daily sports or exercise experience greater benefits than those who do not (Lim et al., 2020; Loudon et al., 2014; Maleki et al., 2025). Studies showed that sports and exercise can improve FMS scores. In this subgroup analysis, both groups showed moderate to high heterogeneity, which may have contributed to heterogeneity in the overall FMS scores analysis.

Region-based subgroup analysis showed no significant differences between both genders, suggesting that cultural and environmental factors may not fully affect FMS scores. High heterogeneity was found in the Europe group, indicating the heterogeneity across studies. Further studies should investigate the age, activity, region-based, and other possible variables to better clarify the differences.

### 4.2 Individual FMS score between genders

#### 4.2.1 Shoulder mobility

This meta-analysis demonstrates a significant gender difference in shoulder mobility (p = 0.01), with females generally exhibited greater shoulder mobility than males. The shoulder mobility test, which evaluates both joint mobility and stability (Cook et al., 2014a; 2014b), is critical in assessing an individual’s ability to attain an appropriate range of motion and maintain stability throughout movement. Joint mobility reflects the ability to attain an appropriate range of motion. However, joint stability is defined as the ability to achieve the correct angular position and the rigidity of the joint, particularly under load (Dill et al., 2014). Previous research has consistently shown that women tend to be more flexible and have a wider extent of movement in their joints, especially in the shoulder (Garcia-Pinillos et al., 2019; Hwang et al., 2024; Moran et al., 2017; Razi, 2016), This heightened range of motion is often linked to differences in joint structures and muscle flexibility in contrast to men.

Generally, there are six movements of a person’s shoulder performed in their daily life routine: adduction and abduction, external and internal rotation, flexion and extension (Gill et al., 2020). These movements are crucial for assessing shoulder mobility in the FMS test. Physiological and structural differences between both genders could contribute to the range and performance of these shoulder movements. A Study showed that the flexion and abduction ranges of shoulder movement decrease with age, with mean reductions of 43° in males and 40.6° in females for flexion, and 39.5° in males and 36.9° in females for abduction (Gill et al., 2020). This suggested that males exhibited a more pronounced reduction in shoulder movement compared to females.

There are three primary bones (clavicle, humerus, and scapula) and four muscles (pectoralis minor, latissimus dorsi, teres major, and subscapularis) involved in the movements of the shoulder. In a study, structural difference was measured across both genders, where a shorter distance between the supraglenoid tubercle and scapular (part of the scapula bone) was found in females compared to males (Mathews et al., 2017). This anatomical variation may influence the soft-tissue flexibility (capsule, ligaments, muscle-tendon units) during the shoulder mobility test. Hormonal differences across both genders may also affect shoulder mobility. Estrogen was found to be higher in females, which was associated with increased ligamentous laxity and joint flexibility (Silke, 2011). These physiological and structural changes could contribute to greater shoulder mobility in females than in males.

Further research has also shown that as the shoulder joints move through their flexion range, shoulder stiffness increases progressively as the joints approach their maximal range of motion (Hwang et al., 2024). This stiffness is often noticeable in males than in females, demonstrating greater resistance to movement as the joint nears its maximal range. Studies have further demonstrated that shoulder hardness indicates that males exhibit greater shoulder stiffness than females (Horobeanu et al., 2022). This finding is significant for the design of sport-specific rehabilitation programs. These sport-specific rehabilitation programs could help with targeted flexibility training in sports like swimming, volleyball, and tennis, where athletes perform more overhead movements. In these sports, male athletes were found to be less flexible in this test, and may require mobility-based interventions to avoid shoulder injuries. For instance, a study involving 43 runner found that females exhibited lower shoulder hardness during the shoulder mobility test (p = 0.018) than males (Loudon et al., 2014). These outcomes support our meta-analysis, which underscores greater shoulder mobility and flexibility in females during the shoulder mobility test.

#### 4.2.2 Active straight-leg raise

The ASLR test is commonly employed to assess the stretching capacity of the hamstrings, gastrocnemius (calf muscle), and soleus (the flat muscle beneath the gastrocnemius), while maintaining thoracic stability. The findings revealed a minor but statistically significant difference (p = 0.002), with females performing better than males. This outcome aligns with prior studies, which consistently show that women generally have larger hamstrings and greater flexibility, both of which are known to elevate ASLR scores (Agresta et al., 2014; Thomas et al., 2022). Research has also revealed the connection between athletic performance (physically) and ASLR movements, noting that these connections vary by gender. In particular, the ASLR test in females shows a stronger positive correlation with core strength, as assessed by sit-ups, than in males.

Despite the gender disparities observed in ASLR, most of these distinctions were evident in joint movement (kinematic parameters) and secondary motion zones, such as the frontal and transverse planes during running mechanics. Studies involving children engaged in running activities have shown that young runners generally suffer most from knee joint pathology, which can result in lower extremity injuries (Loudon et al., 2014). This underscored the need to consider gender disparities in movement mechanics. Females exhibit markedly elevated peak angles of hip adduction, inner rotation, and knee abduction, which have been consistently observed (Sakaguchi et al., 2014), further highlighting the specific movement pattern between genders.

Additionally, a study with 43 runner athletes and another with 200 NCAA division female athletes has demonstrated significant gender differences in ASLR and SM, showing that females generally performed better in these tests compared to males (Duncan et al., 2013; Loudon et al., 2014).^,^ Under typical loading conditions, females tend to have lower hamstring stiffness than males, consistent with earlier studies indicating that women generally exhibit greater hamstring flexibility. This increased flexibility, which contributes to higher ASLR scores, underscores the importance of mobility training for both genders and underscores the significance of flexibility in overall movement performance (Johnson et al., 2023). Male athletes could benefit from preventing muscle strain injuries by improving hamstring and calf flexibility through mobility-based training programs. Core stability exercises may help female athletes integrate their natural flexibility with strength, reducing the risk of injuries caused by instability or overuse.

Due to the substantial heterogeneity in the analysis, a sensitivity analysis was conducted. The studies by Samer et al. (2024), Wu et al. (2021) both reported heterogeneity. The Samer et al. (2024) study was conducted on the Palestinian population, making it the only study to focus on this specific demographic. The heterogeneity could be attributed to cultural and traditional differences compared to the other included studies. On the other hand, the heterogeneity in Wu et al. (2021) has been explained in the overall FMS test analysis between males and females. After conducting a sensitivity analysis, heterogeneity was reduced to 46%.

#### 4.2.3 Hurdle step

The purpose of HS is to evaluate lower limb joint mobility in the sagittal plane, assessing how well an individual can move the lower limb through a proper range of motion while maintaining proper stability. The results of this analysis favor females’ p = 0.01, suggesting that males exhibit greater asymmetries in this test, suggesting males may have decreased lower limb mobility in the sagittal plane compared to females. These findings align with previous studies that highlight how females often adopt a more vertical posture in the sagittal plane when contacting the ground, demonstrating greater knee flexion and improved mobility (Decker et al., 2003).

The challenges associated with the hurdle step may arise from inadequate stability in the stance leg or insufficient mobility in the stepping leg, which reflects postural instability, particularly in males during this task (Cook et al., 2006a). Postural stability plays a key role in the hurdle step test, which requires the coordination of trunk muscles and joints, particularly the hips, knees, and ankles, moving through a controlled range of motion. In the stance leg, without adequate stability, people are more prone to suffer compensation for this task. This finding indicates gender differences in lower limb joint mobility and stability, which may affect overall functional movement scores between both genders. These findings align with studies that emphasize significant differences (García-Luna et al., 2020). As Duncan et al. (2013) conducted an FMS assessment on 7- to 10-year-old normal-weight and obese children, observed that females performed better than males on both the HS and ASLR tests. This reinforces the idea that females are better at this movement pattern task, which requires mobility and stability, as is evident from a young age. This highlights the importance of lower extremity mobility and joint stability training programs for male athletes to help prevent injuries like ACL tears, which are often produced during sports that require sudden stops, pivoting, changes in direction, and dynamic lower-limb movements. Further research is needed to investigate gender differences in this task while accounting for individual weight and age.

#### 4.2.4 Rotary stability

RS is associated with core stability in the horizontal plane, requires neuromuscular control to improve lower-limb functional movements, facilitates energy transfer between body segments through the torso, and requires sufficient stability in the transverse plane (Cook et al., 2006a). Our analysis demonstrated significantly higher RS scores in females. Males who score lower on the test may have less control over their hip, pelvic, and trunk muscles, which may contribute to inappropriate lower-limb mechanics (Cook et al., 2006b). This aligns with previous studies showing that males have less hip flexion and a smaller frontal pelvic angle than females (Czuppon et al., 2017; Mier and Shapiro, 2013), which may affect their performance on this task. Mitchell et al. found that FMS test females exhibited fewer asymmetries than males when performing the FMS test, contributing to enhanced coordination and stability (Mitchell et al., 2015). Improved rotary stability is associated with better lower extremity coordination, suggesting that females may have an advantage in this domain due to superior neuromuscular control (Hu et al., 2023) or greater movement efficiency (Bond et al., 2014). The increased hip range of motion, possibly influenced by joint laxity and hormonal factors, may further enhance this advantage by enabling females to execute more effective movement patterns during rotational tasks. These findings suggest that male athletes should prioritize core stability training to enhance core engagement and neuromuscular control and to mitigate core-related injuries. Female athletes should focus on strength training to maintain their inherent flexibility, further optimize dynamic coordination, and prevent injuries.

A sensitivity study was conducted to mitigate heterogeneity. Excluding the research by Boguszewski et al. (2017), García-Luna et al. (2020), heterogeneity was reduced to 48%. Since rotary stability requires core stability and coordination, athletes in these studies (Judo and Hockey players) may perform differently based on their specific physical demands (Boguszewski et al., 2017). focused primarily on hockey and non-hockey players. In this analysis, we included only results from hockey players, resulting in an imbalanced sample (5 girls and 33 boys). This imbalanced sample size could be a contributing factor to the observed heterogeneity.

#### 4.2.5 Trunk stability

The TSPU was correlated with abdominal stability and strength, especially torso stability in the sagittal plane. This study indicates that men, on average, outperformed women in the trunk stability test, with statistically significant difference (z = 3.37, p = 0.0007), suggesting that males generally possess superior upper limb strength and trunk stabilization in dynamic conditions. These findings align with previous studies suggesting that males exhibit greater lumbar flexion than females during straight trunk flexion (Mier and Shapiro, 2013). These differences underscore the need to consider gender-based differences while analyzing the impact of core stability on spinal kinematics. The difference suggested that men had a better ability to maintain trunk stability during dynamic motions, which is crucial for many physical activities.

Core stability plays an important role in performing the trunk stability test. Four primary muscles, the transversus abdominis, diaphragm, pelvic floor muscle, and multifidus work together to maintain a rigid and stable trunk during the TSPU (Hibbs et al., 2008). However, there may be gender-based differences in both the size and ability to stretch these muscles, which can impact performance (Czuppon et al., 2017). For example, females were found to have wider pelvis (Hedt et al., 2022), which affects the alignment of core muscle attachments, making it more difficult to maintain straight trunk alignment. A broader pelvic structure may result in an increased lumbar spine tilt, complicating the retention of appropriate alignment during dynamic movement (Mier and Shapiro, 2013). A study found that males demonstrated greater co-activation of trunk and hip muscles, suggesting they have significant advantages in core stability tasks (Ireland et al., 2012).

Additionally, Physiological differences can affect core stability and FMS scores in both genders. The study found that females tend to have greater joint laxity and lower testosterone levels compared to males (Gordon et al., 2013). This could result in lower core stiffness in females (Gomes et al., 2022), reducing their ability to produce more power during movements that require core stability and activation compared to males.

Research suggests that women generally exhibit lower core stability, which may affect performance in activities that require stronger core stability for optimal movement (Chimera et al., 2015; Sharrock et al., 2011). These differences in core stability might be due to variations in core muscular activation instead of strength. These gender differences in performance may be partially explained by the complex, movement-based, and multidimensional characteristics of FMS testing, as well as by the definition and assessment of core stability (Willson et al., 2005). These results emphasize the need to customize the training programs to address the core stability requirements of both genders.

Further supporting this notion (Picco et al., 2018), found that females demonstrated less scapular posterior tilt than males, which may have contributed to differences in upper-body movement mechanics. Similarly Szucs and Borstad (2013), reported gender-based differences in trapezius muscle activation during certain physical activities. These differences in muscle activation during activities tend to be more challenging for female athletes than for their male counterparts during the trunk stability push-ups test, possibly due to differences in muscle recruitment and core strength (Boucher et al., 2021). These findings suggest that female athletes should focus on targeted strengthening exercises to bridge the gap in core and upper body strength. This will help improve performance and reduce musculoskeletal injuries.

Lastly, heterogeneity I^2^ = 76% was observed in this analysis, and to address this issue, a sensitivity analysis was conducted. Notably, three studies (Razi, 2016; Vehrs et al., 2021; Wu et al., 2021) contributed to the heterogeneity. The Wu et al. (2021) study stands out because it is the only one among the 12 studies to focus on 7- to 10-year-old participants, specifically from China. This demographic could introduce heterogeneity into this analysis due to factors such as fitness level, age differences, and cultural differences. The study by Vehrs et al. (2021) focused on different weight categories, such as normal weight, overweight, and obese children, while also considering the developmental stage of participants. In this study, 55% of boys and 58% of girls exhibited asymmetries in the core stability required test. This variation in body composition, as well as children developmental stage, could increase heterogeneity in the analysis. Finally, the study by Razi (2016) focused on taekwondo athletes, a population with specific skills and training, distinguishing them from other groups in the analysis, which may further heterogeneity.

Furthermore, the research participants had a history of injuries, which may have impacted their performance on the FMS exam. Including athletes with prior injuries may increase heterogeneity due to potential physical restrictions or compensatory movements. After conducting the sensitivity analysis, heterogeneity was reduced to I^2^ = 47%, suggesting that these studies significantly influenced the overall heterogeneity across studies.

### 4.3 Non-significant (DS and ILL)

The squat is a key exercise often used in physical training, primarily to enhance athletic performance, especially in weightlifting and powerlifting (ESCAMILLA et al., 2001; Schoenfeld, 2010). This meta-analysis examined variations in gender differences in scores for the deep squat (DS) and in-line lunge (ILL), along with other tests discussed earlier. The findings showed no significant gender differences in these functional mobility tasks, indicating that both genders perform similarly in these functional movement assessments. The deep squat and in-line lunge require flexibility, mobility, and stability in several joints and muscles, including the ankles, knees, hips, shoulders, quadriceps, hamstrings, and feet. Flexibility in these joints is crucial for optimal performance. However, not only is the flexibility important, but also the coordination of strength and mobility to stabilize the trunk and lower limbs during movement.

Despite the importance of these factors, no significant gender differences were observed in these multi-joint tasks. This may be due to the balance between greater core and lower body strength in males and superior mobility and flexibility in females, which may compensate for potential differences in both DS and ILL tests. For example, mobility and flexibility may have facilitated better squat depth, while strength may have supported proper alignment and balance during both the squat and lunge tests. AS a result, these physiological differences may have counterbalanced each other, resulting in no significant gender difference in performance, consistent with the findings of our meta-analysis.

These results may be attributed to the universal neuromotor demands of bilateral squatting and lunging, which are influenced by individual physical characteristics, rather than gender differences (Demers et al., 2018; Gomes et al., 2022). Specifically, leg length ratios, particularly femoral and tibia lengths, which significantly impact hip and knee flexion, play a crucial role in the movements required in the DS and ILL tests. While individual studies report gender differences, two studies report higher female performance in the DS test (García-Luna et al., 2020; Wu et al., 2021). One study in the ILL test (Boguszewski et al., 2017) showed a difference compared to males, while another study from both tests favored males over females (Razi, 2016; Vernetta-Santana et al., 2019). However, the overall analysis revealed no substantial difference (p > 0.05).

The research conducted by Johnson et al. (2023) involving 93 ROTC cadets found no significant sex differences in DS (p = 0.865). In contrast, our findings align with those reporting no significant variations between sexes in these tests (Abraham et al., 2015). Additionally, the study by Mitchell et al. (2015) concluded that both males and females exhibited equal asymmetries during the physical performance of these tests. The findings indicate that the tests aim to evaluate athletes’ overall movement quality rather than solely specific gender-based strength or flexibility elements (Kim et al., 2021). Since both males and females perform similarly in these bilateral exercises that require symmetrical movements, this implies that flexibility, mobility and stability training should focus on improving overall quality movement regardless of gender differences observed in other FMS individual tests. These training programs emphasize improving joint alignment, posture, and muscle activation, which may help reduce the risk of injuries. This further strengthens the idea that in these specific functional movements, gender does not significantly influence performance.

Deep squat study showed 66% heterogeneity, whereas in-line lunge analysis showed 58%. A sensitivity analysis was performed for both tests to address this. In deep squat analysis, excluding the research by García-Luna et al. (2020), Wu et al. (2021), the heterogeneity was reduced to 40%. The heterogeneity observed in these studies may be attributed to Significant differences they reported in the deep squat test, particularly favoring females, which could contribute to the heterogeneity. Therefore, the disparities between the two studies may have substantially influenced the outcomes.

Conversely, the analysis of the in-line lunge tests, a study by Vernetta-Santana et al. (2019), revealed substantial heterogeneity across studies. The findings of this study differed from those of the other included studies, demonstrating a pronounced difference between the both genders, favoring males. This was not observed in the other studies and is likely contributing to the heterogeneity, potentially leading to misinterpretation of the overall result of this analysis. After the sensitivity analysis, the results shifted to favor females (p = 0.01) in the in-line lunge test. One possible reason for this discrepancy was the relatively small and imbalanced sample size in the Vernetta-Santana et al. (2019) study (11 males and 24 females), which could have led to heterogeneity in analysis.

## 5 Conclusion

This meta-analysis provides specific information about gender differences in FMS scores, revealing distinct movement patterns between males and females. The results indicated that females had higher functional movement capacity, as reflected in the overall FMS composite scores. However, gender differences were more prominently observed in specific FMS components. Females performed better in active shoulder mobility, straight leg-raise test, hurdle step, and rotary stability, reflecting increased flexibility, stability, and mobility. In contrast, a significant difference was observed in the trunk stability push-up test, indicating greater core stability and strength. Interestingly, no significant results were observed in the deep squat and in-line lunge tests. Subgroup analysis revealed significant effects of younger age and sports participation, underscoring the crucial roles of age and sports participation in FMS score performance. However, no significant difference was found in the region-based subgroup. The results suggested that the effects of age and sports participation on FMS scores vary by age and sports activity. Future research should focus on underlying attributes that contribute to these gender differences, examine the role of fat mass and training volume, and further address inconsistencies observed in the deep squat and in-line lunge tests.

### 5.1 Strengths and limitations

This meta-analysis incorporated the latest studies to analyze overall FMS and individual FMS test scores, providing a more comprehensive and nuanced evaluation of movement patterns across genders. By including the latest studies, this study provides additional insight into functional movement ability across genders, which may guide subsequent interventions and practices. One key attribute of this analysis is the inclusion of a standardized risk-of-bias assessment and the integration of advanced statistical methods, including funnel plots, Egger’s-test, and trim-and-fill analysis, which enhance the reliability of the findings and methodology. These tools are critical for reporting publication bias and for conducting sensitivity analyses, thereby enhancing the reliability of the results. However, it is important to note that these methods may be less reliable when applied to small samples, as small-study effects may not be fully captured.

This meta-analysis revealed several limitations. Firstly, the study excludes a specific age range and population, except for school children and athletes, which may limit the generalizability of the results to older adults, sedentary individuals, or populations with different levels of physical activity. Moreover, regarding selection bias, the majority of research fails to disclose non-respondents and methods for sample justification; this may also constrain this study. Secondly, most studies are cross-sectional, which may limit the ability to establish links between variables. As cross-sectional studies are collected at a single point in time, they can only show association rather than variable changes over time. These studies may also have lacked information on interventions, such as exercise type, frequency, intensity, and volume, which could have hindered the assessment of long-term effects on FMS scores. Future studies should adopt a longitudinal design to examine how other variables (such as exercise type, frequency, intensity, and volume) change over time and affect FMS scores in both genders. Moreover, this study generalized from non-injured students and athletes, specifically focused on a single age group (6–30 years), which also limits the study.

Additionally, there is also a need to focus on participant activity level, considering gender differences, and explore how specific exercise regimens, such as aerobic and resistance, and flexibility exercise, could impact FMS score between males and females. These factors were not evaluated in this analysis; therefore, by addressing these factors, future studies may provide more precise and customized suggestions for improving functional mobility across genders.

## Data Availability

The original contributions presented in the study are included in the article/Supplementary Material, further inquiries can be directed to the corresponding authors.
