# Supplementary material for: Evaluation of both overall and individual FMS components results in male and female groups: a systematic review and meta-analysis
Source: Front Physiol. 2026 Jan 12;16:1669967. doi: 10.3389/fphys.2025.1669967 (PMC12832502; doi:10.3389/fphys.2025.1669967)
Supplement: Supplementary file 1 [file Table1.DOCX]

# **Search Strategy**

# **PubMed**

| **Search Number** | **Search Query** |
| --- | --- |
| **1** | (((((((functional movement[Title/Abstract]) OR (FMS[Title/Abstract])) OR (Movement quality[Title/Abstract])) OR (Movement pattern[Title/Abstract])) OR (FMS score[Title/Abstract])) OR (functional screening[Title/Abstract])) OR (movement screening[Title/Abstract])) OR (functional movement screening[Title/Abstract]) |
| **2** | ((((((((individual fms[Title/Abstract])) OR (deep squat[Title/Abstract])) OR (hurdle step[Title/Abstract])) OR (shoulder mobility[Title/Abstract])) OR (active straight leg raise[Title/Abstract])) OR (trunk stability push up[Title/Abstract])) OR (in line lunge[Title/Abstract])) OR (in-line lunge[Title/Abstract])) OR (rotary stability[Title/Abstract])) OR (DS test[Title/Abstract])) OR (SM test[Title/Abstract])) OR (HS test[Title/Abstract])) OR (TSPU test[Title/Abstract])) OR (RS test[Title/Abstract])) OR (ASLR test[Title/Abstract])) OR (ILL test[Title/Abstract]) |
| **3** | ((((((((((Gender difference[Title/Abstract]) OR (sex difference[Title/Abstract])) OR (male[Title/Abstract] AND female[Title/Abstract])) OR (male[Title/Abstract])) OR (female[Title/Abstract])) OR (school students[Title/Abstract])) OR (athletes[Title/Abstract])) OR (college students[Title/Abstract])) OR (university students[Title/Abstract])) OR (professional players[Title/Abstract])) OR (sports group[Title/Abstract]) |
| **4** | ((((((((functional movement[Title/Abstract]) OR (FMS[Title/Abstract])) OR (Movement quality[Title/Abstract])) OR (Movement pattern[Title/Abstract])) OR (FMS score[Title/Abstract])) OR (functional screening[Title/Abstract])) OR (movement screening[Title/Abstract])) OR (functional movement screening[Title/Abstract])) AND (((((((((individual fms[Title/Abstract])) OR (deep squat[Title/Abstract])) OR (hurdle step[Title/Abstract])) OR (shoulder mobility[Title/Abstract])) OR (active straight leg raise[Title/Abstract])) OR (trunk stability push up[Title/Abstract])) OR (in line lunge[Title/Abstract])) OR (in-line lunge[Title/Abstract])) OR (rotary stability[Title/Abstract])**)** OR (DS test[Title/Abstract])**)** OR (SM test[Title/Abstract])**)** OR (HS test[Title/Abstract])**)** OR (TSPU test[Title/Abstract])**)** OR (RS test[Title/Abstract])**)** OR (ASLR test[Title/Abstract])**)** OR (ILL test[Title/Abstract])**)** |
| **5** | ((((((((functional movement[Title/Abstract]) OR (FMS[Title/Abstract])) OR (Movement quality[Title/Abstract])) OR (Movement pattern[Title/Abstract])) OR (FMS score[Title/Abstract])) OR (functional screening[Title/Abstract])) OR (movement screening[Title/Abstract])) OR (functional movement screening[Title/Abstract])) AND (((((((((individual fms[Title/Abstract])) OR (deep squat[Title/Abstract])) OR (hurdle step[Title/Abstract])) OR (shoulder mobility[Title/Abstract])) OR (active straight leg raise[Title/Abstract])) OR (trunk stability push up[Title/Abstract])) OR (in line lunge[Title/Abstract])) OR (in-line lunge[Title/Abstract])) OR (rotary stability[Title/Abstract])**)** OR (DS test[Title/Abstract])**)** OR (SM test[Title/Abstract])**)** OR (HS test[Title/Abstract])**)** OR (TSPU test[Title/Abstract])**)** OR (RS test[Title/Abstract])**)** OR (ASLR test[Title/Abstract])**)** OR (ILL test[Title/Abstract])**)** AND (((((((((((Gender difference[Title/Abstract]) OR (sex difference[Title/Abstract])) OR (male[Title/Abstract] AND female[Title/Abstract])) OR (male[Title/Abstract])) OR (female[Title/Abstract])) OR (school students[Title/Abstract])) OR (athletes[Title/Abstract])) OR (college students[Title/Abstract])) OR (university students[Title/Abstract])) OR (professional players[Title/Abstract])) OR (sports group[Title/Abstract])) |

# **Web of Science**

| **Search number** | **Search query** |
| --- | --- |
| **1** | **(((((((TI=(functional movement)) OR TI=(FMS)) OR TI=(Movement quality)) OR TI=(Movement pattern)) OR TI=(FMS score)) OR TI=(functional screening)) OR TI=(movement screening)) OR TI=(functional movement screening)** |
| **2** | (((((((((((((((TI=(individual fms)) OR TI=(deep squat)) OR TI=(hurdle step)) OR TI=(shoulder mobility)) OR TI=(active straight leg raise)) OR TI=(trunk stability push up)) OR TI=(in line lunge)) OR TI=(in-line lunge)) OR TI=(rotary stability)) OR TI=(DS test)) OR TI=(SM test)) OR TI=(HS test)) OR TI=(TSPU test)) OR TI=(RS test)) OR TI=(ASLR test)) OR TI=(ILL test) |
| **3** | ((((((((((TI=(Gender difference)) OR TI=(sex difference)) OR TI=(male and female)) OR TI=(male)) OR TI=(female)) OR TI=(school students)) OR TI=(athletes)) OR TI=(college students)) OR TI=(university students)) OR TI=(professional players)) OR TI=(sports group) |
| **4** | #1 OR #2 |
| **5** | #4 AND #3 |

# **Google Scholar**

| **Search** | **Search Strategy** |
| --- | --- |
|  | **functional movement screening OR FMS OR Movement quality OR Movement pattern OR FMS score OR functional screening OR movement screening OR functional movement OR individual fms OR deep squat OR hurdle step OR shoulder mobility OR active straight leg raise OR trunk stability push up OR in line lunge OR in-line lunge OR rotary stability OR DS test OR SM test OR HS test OR TSPU test OR RS test OR ASLR test OR ILL test AND Gender difference OR sex difference OR male and female OR male OR female OR school students OR athletes OR college students OR university students OR professional players OR sports group** |
