# Supplementary material for: Evaluation of both overall and individual FMS components results in male and female groups: a systematic review and meta-analysis
Source: Front Physiol. 2026 Jan 12;16:1669967. doi: 10.3389/fphys.2025.1669967 (PMC12832502; doi:10.3389/fphys.2025.1669967)

**Evaluation of Overall FMSTM and Individual Test Results in Male and Female Groups: A Systematic Review and Meta-Analysis**

# **Overall FMS^TM^ Score between gender**

## **Sensitivity Analysis**


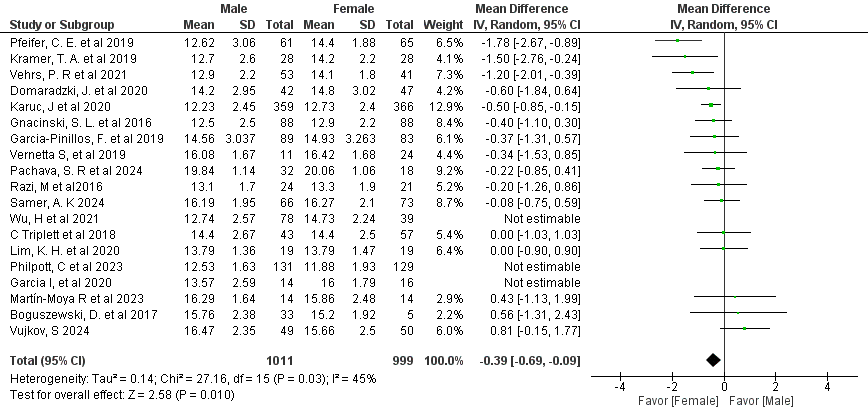


## **Egger’s Regression analysis**


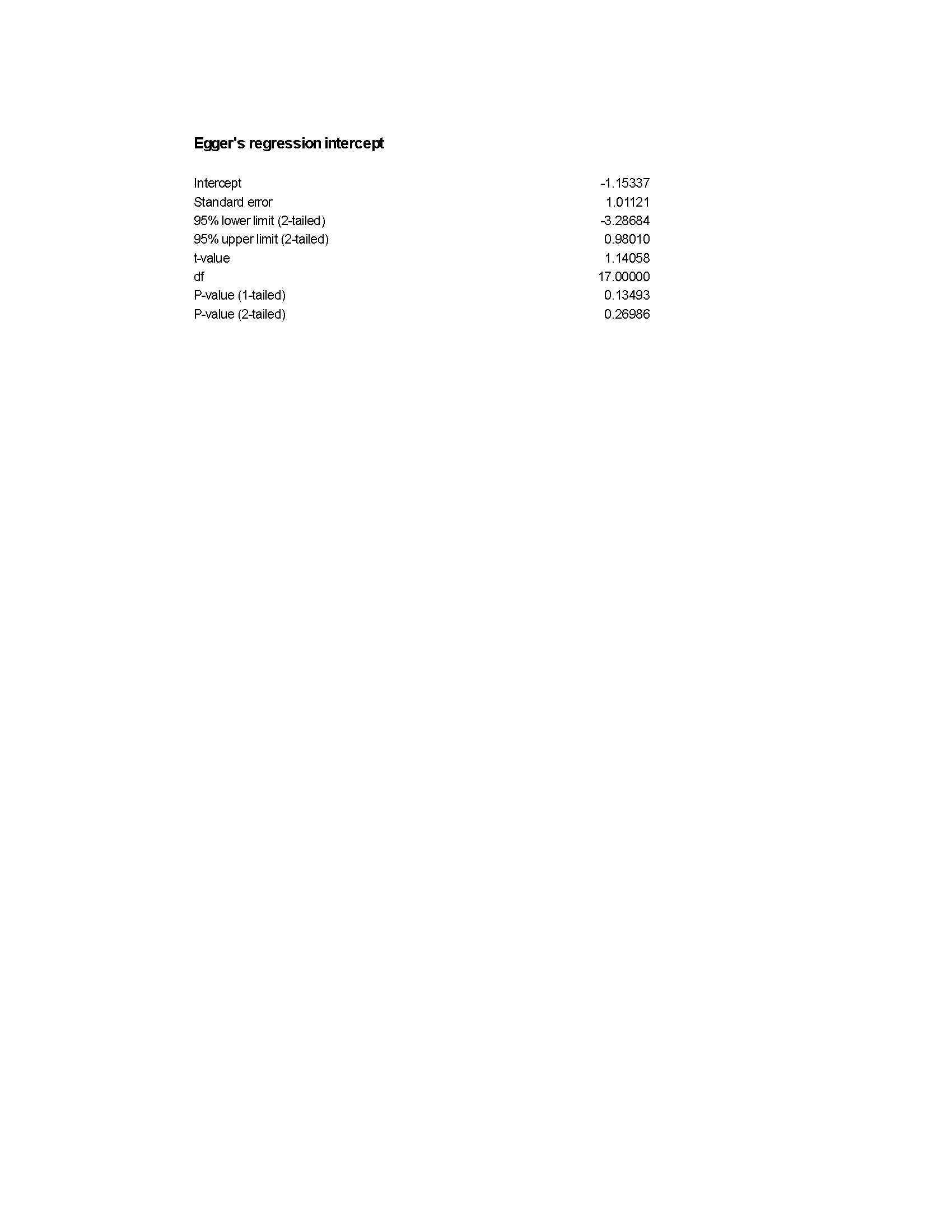


**Subgroup analysis**

**Age forest plot**

**
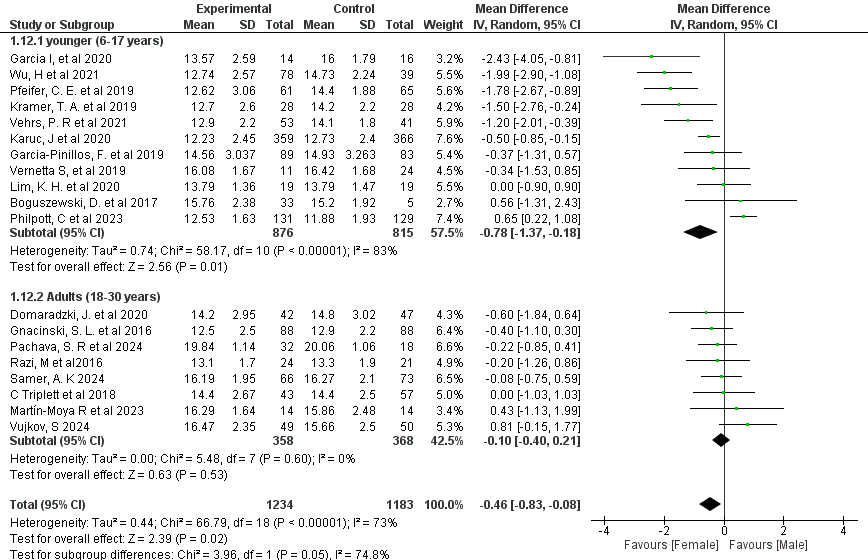
**

**Sports Participation**

**
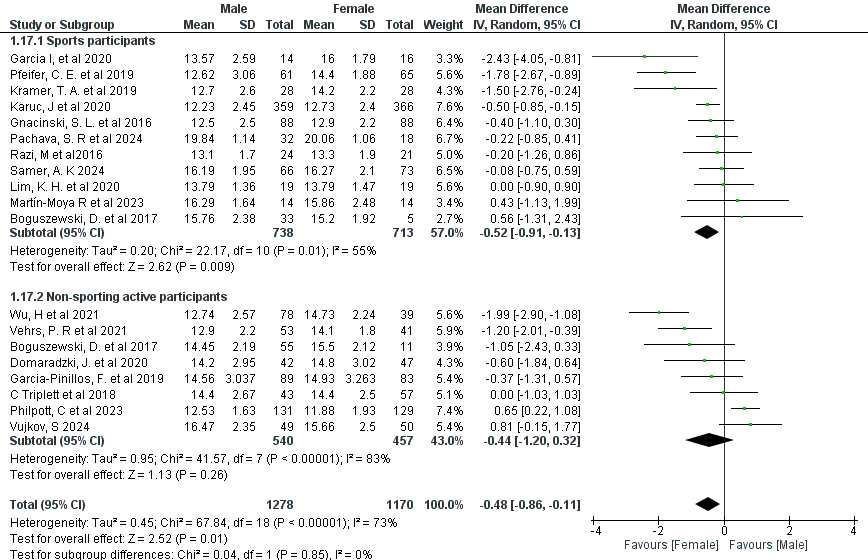
**

**Region-based**

**
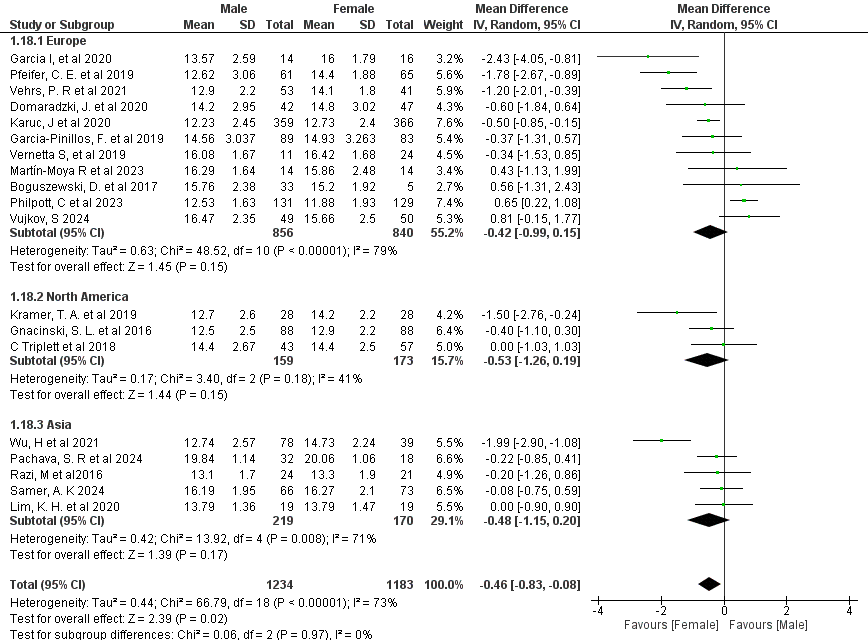
**

# **Individual FMSTM score between gender**

## **Shoulder mobility**

### **Egger’s Regression**


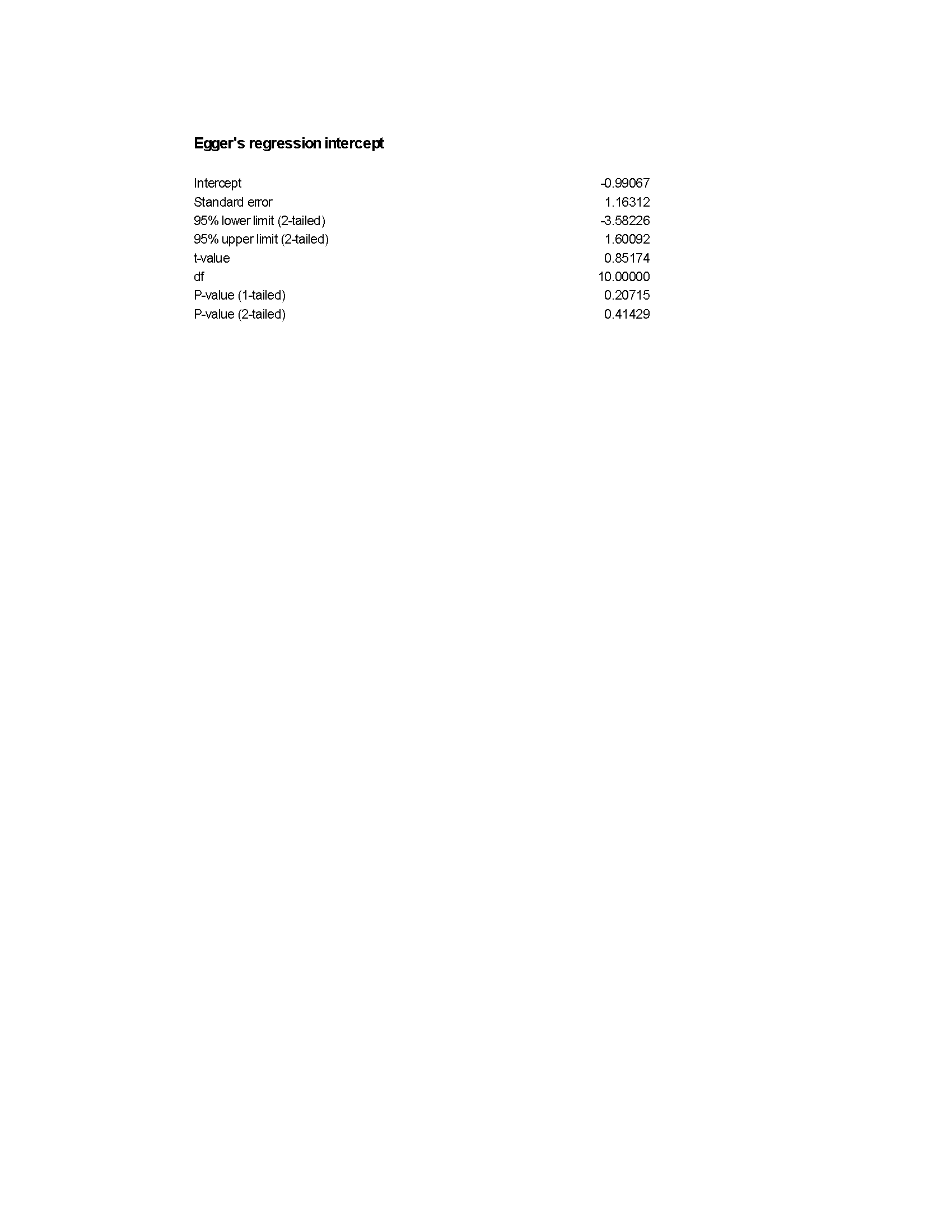


## **Active straight leg raise test**

### **Sensitivity Analysis**


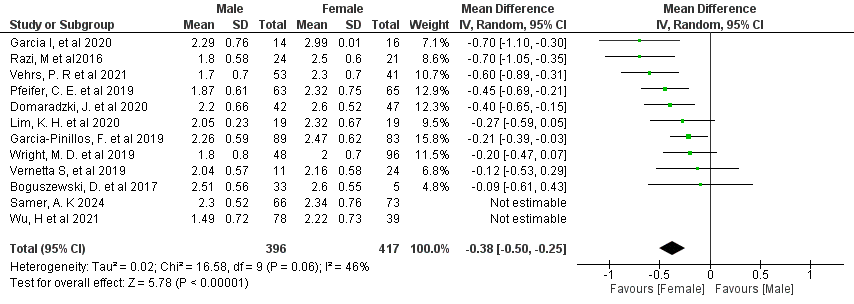


### **Egger’s regression**


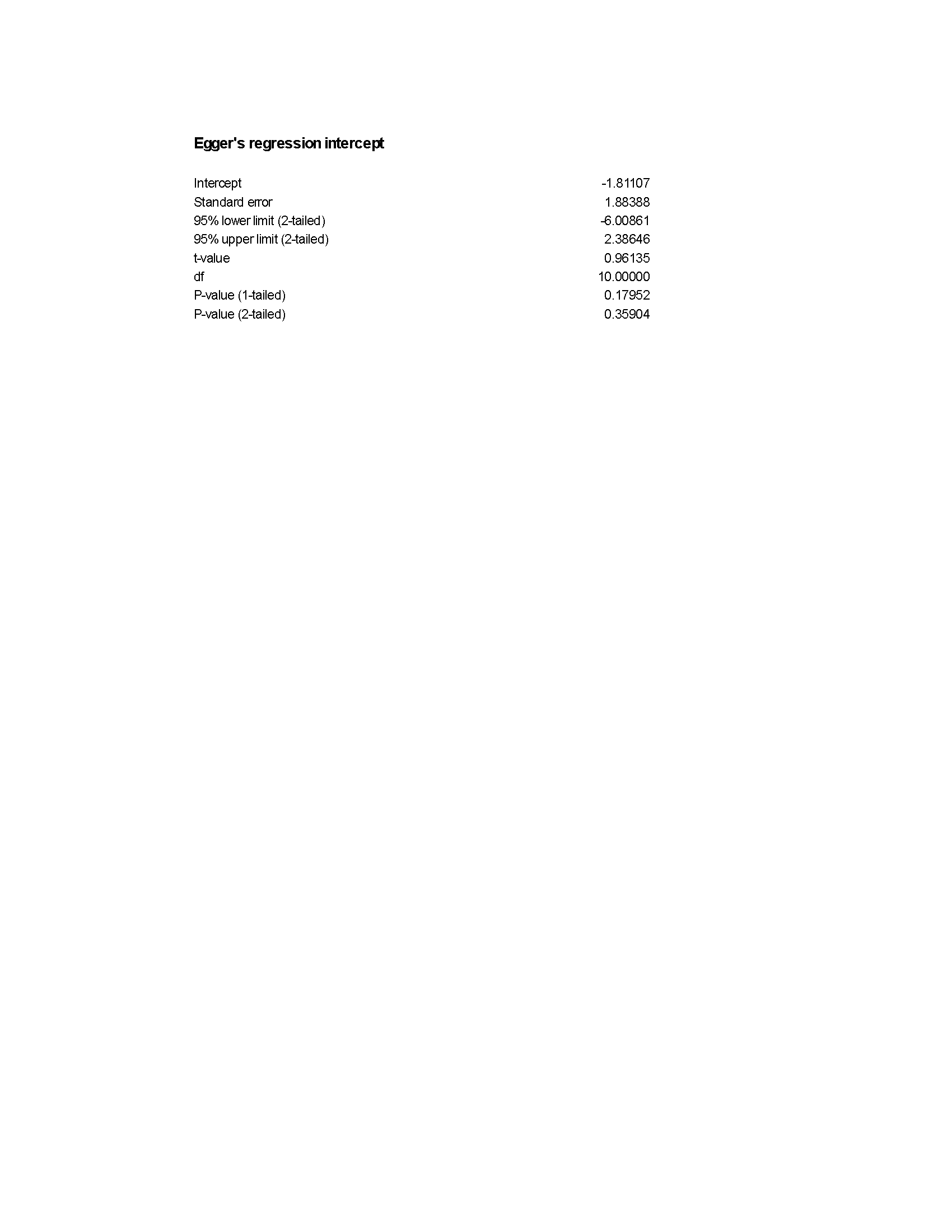


## **Hurdle step test**

### **Egger’s regression**


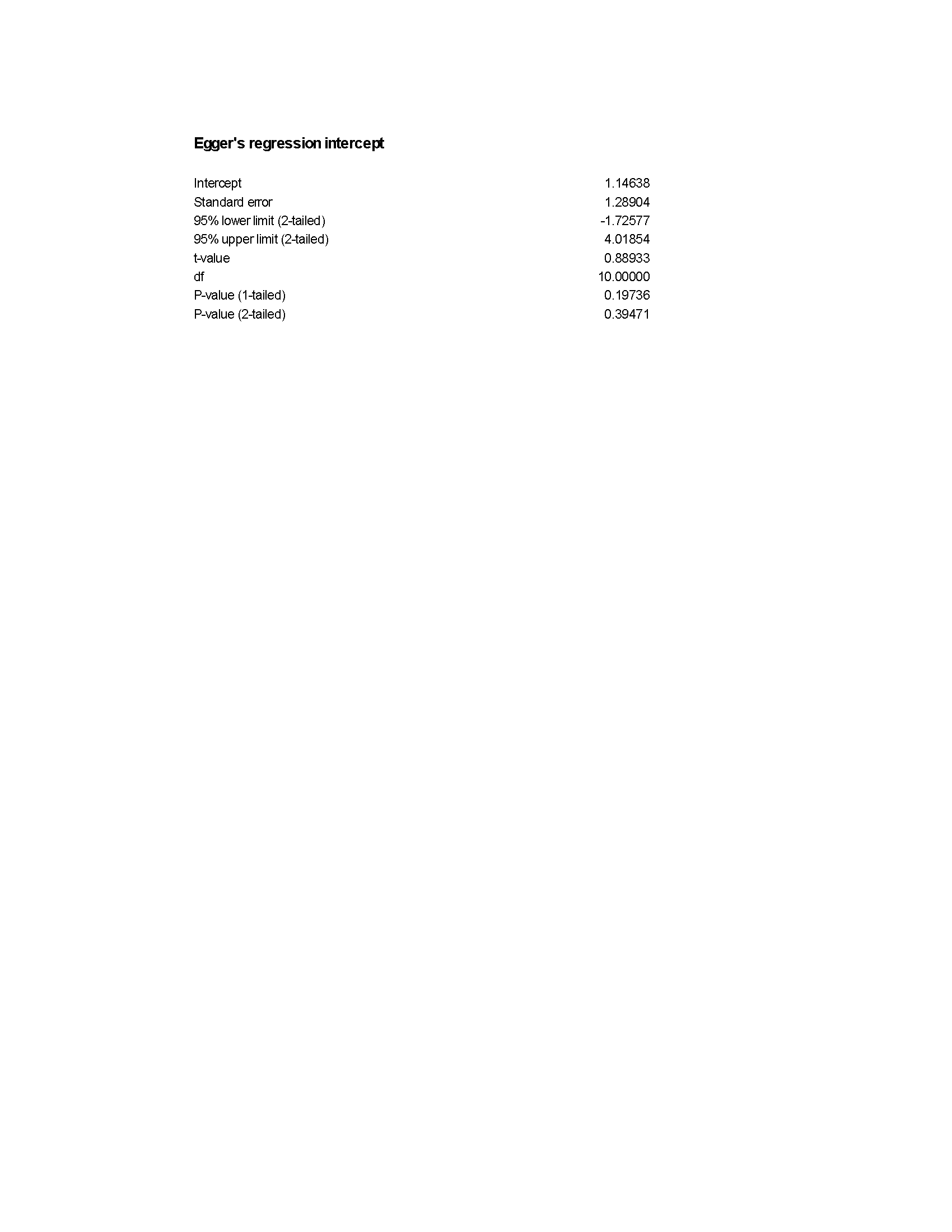


## **Rotary stability test**

### **Sensitivity Analysis**


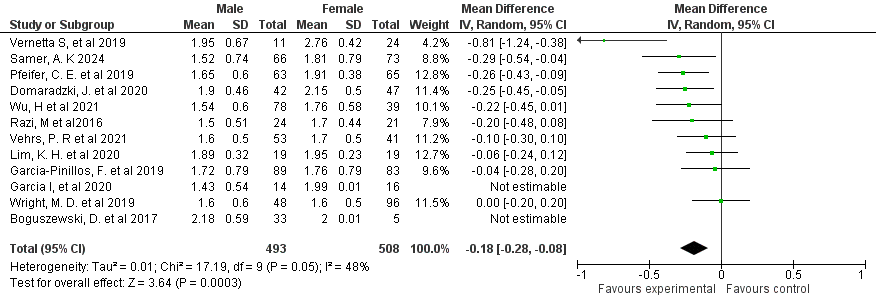


### **Egger’s regression**

**
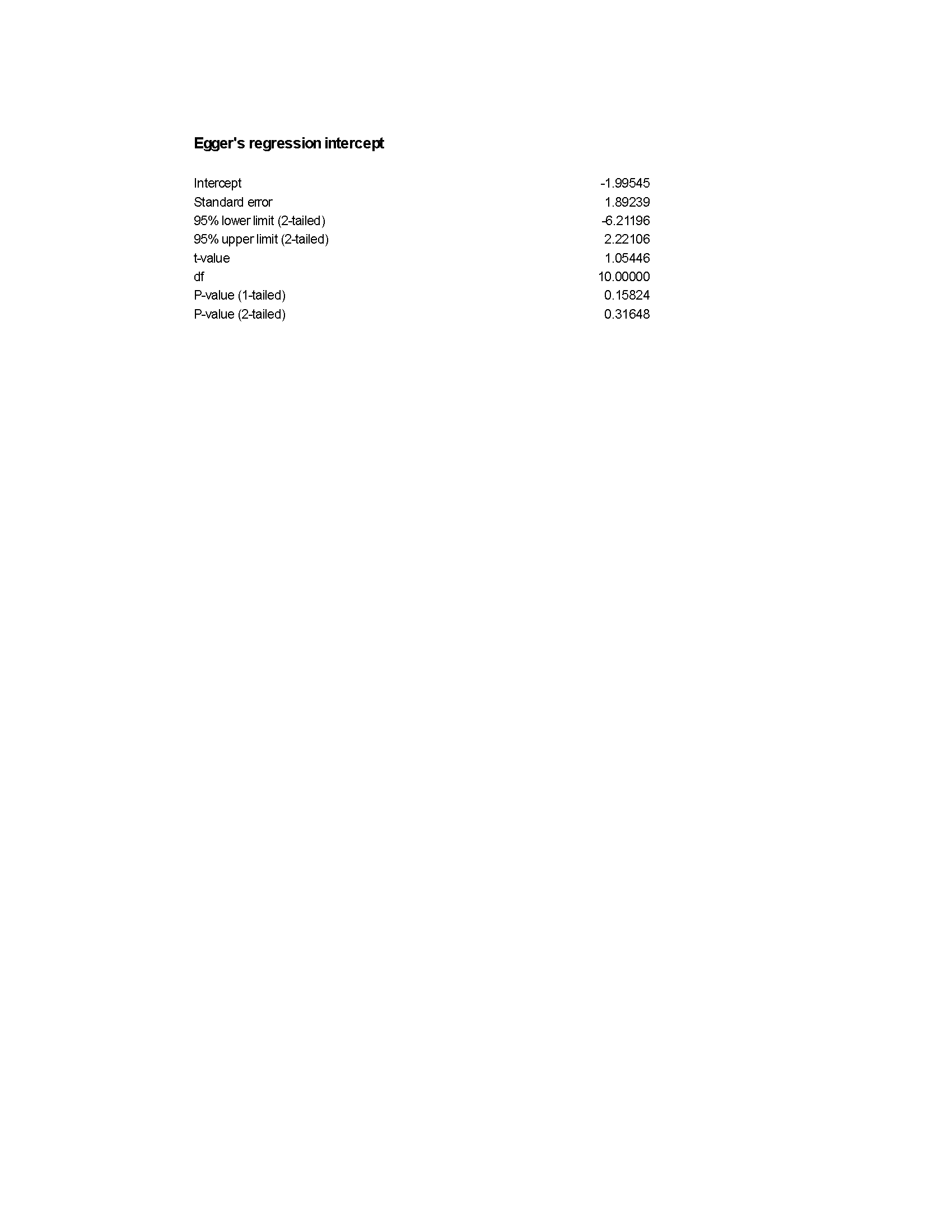
**

## **Trunk stability push-up test**

### **Sensitivity Analysis**


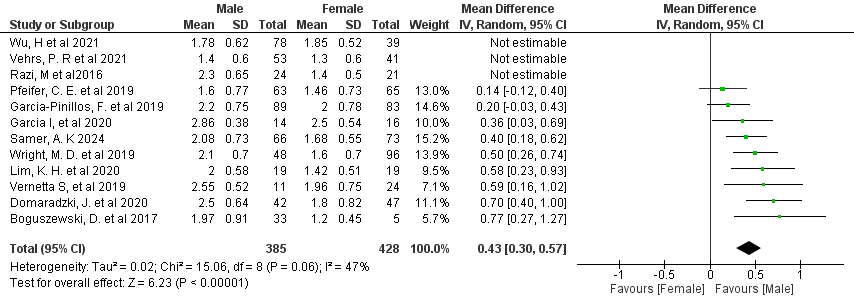


### **Egger’s Regression analysis**

**
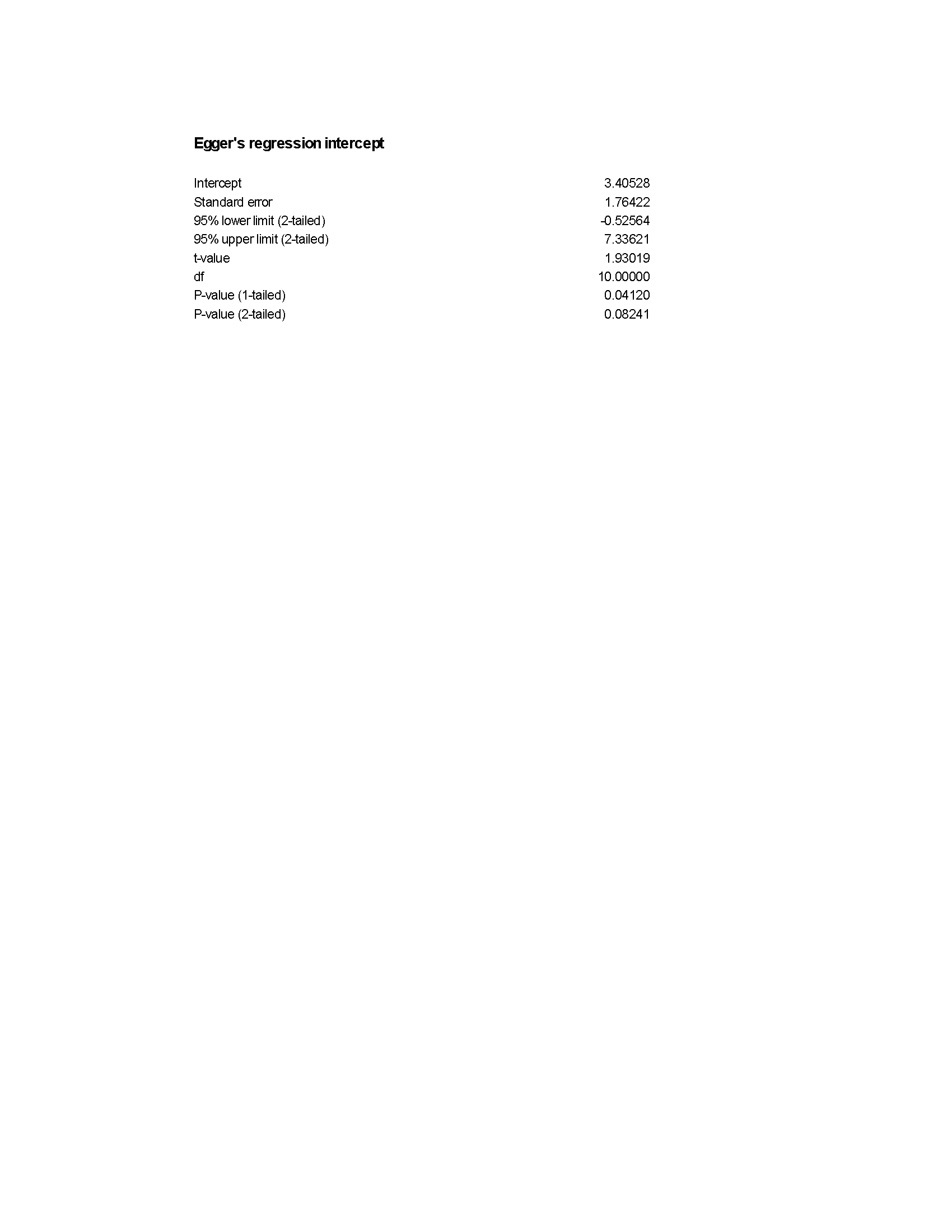
**

### **Trim and Fill analysis**


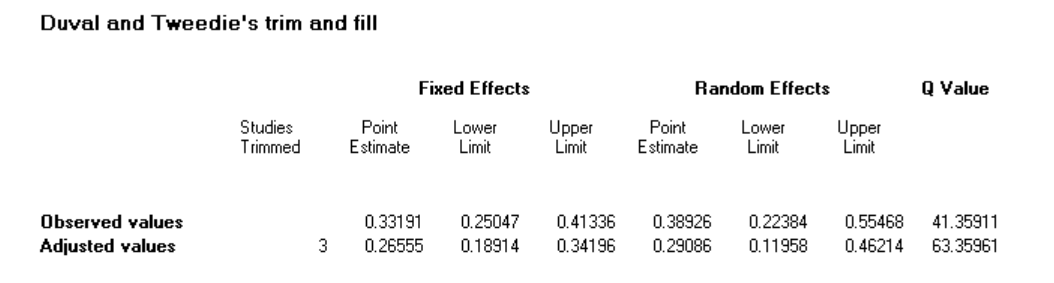


## **Deep squat test**

### **Sensitivity Analysis**


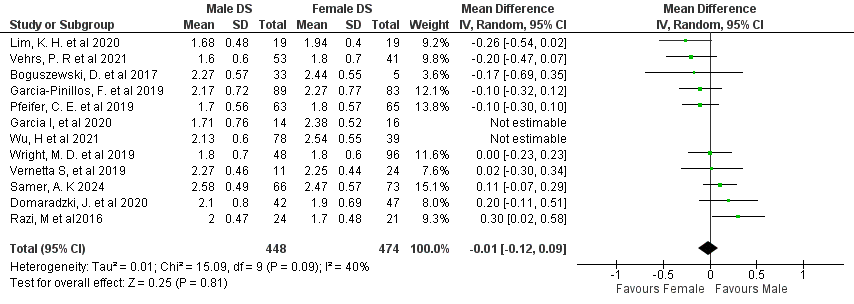


### **Egger’s regression**

**
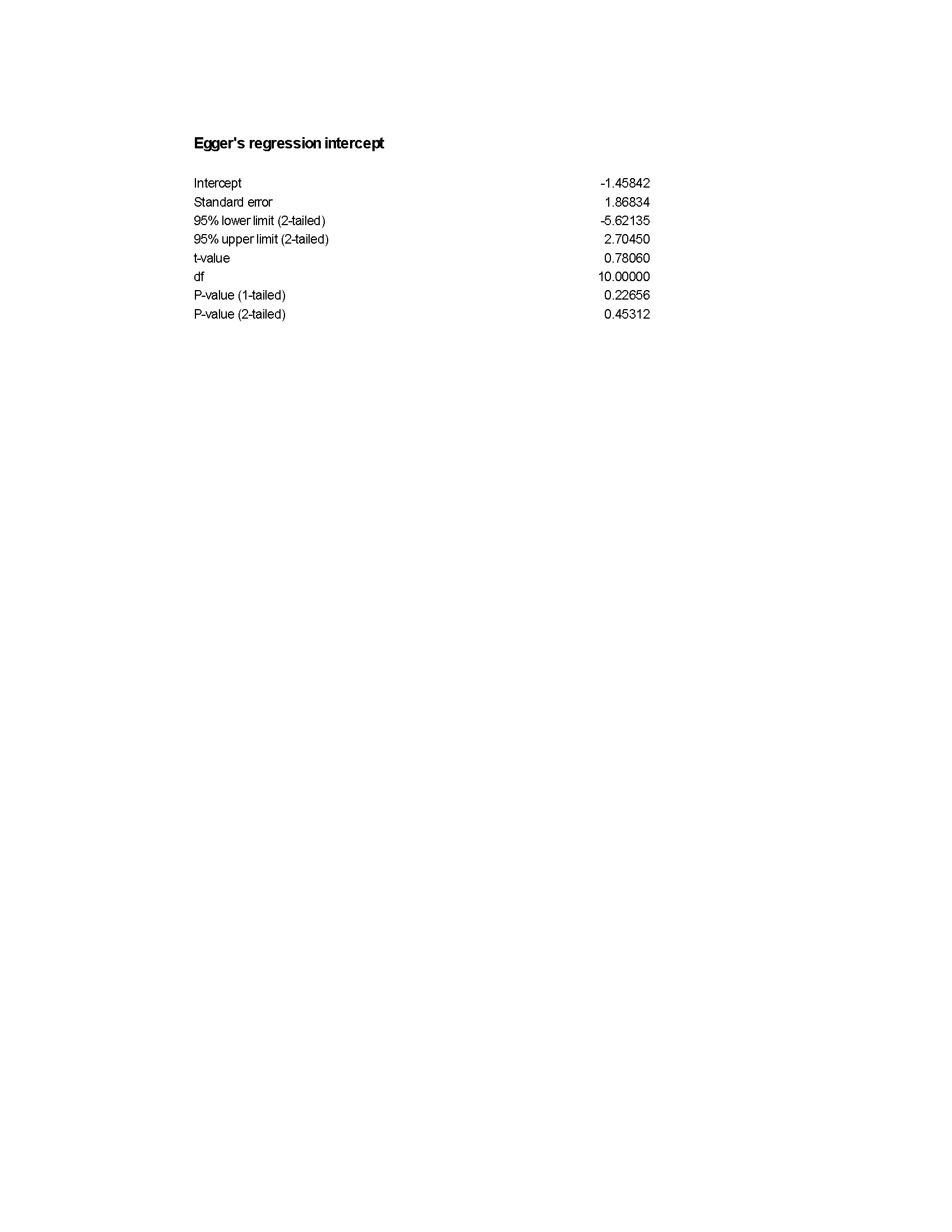
**

## **In-Line Lunge test**

### **Sensitivity Analysis**

###
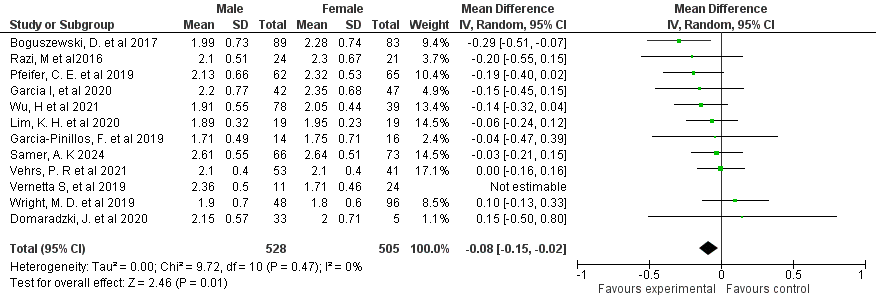
 **Egger’s Regression**


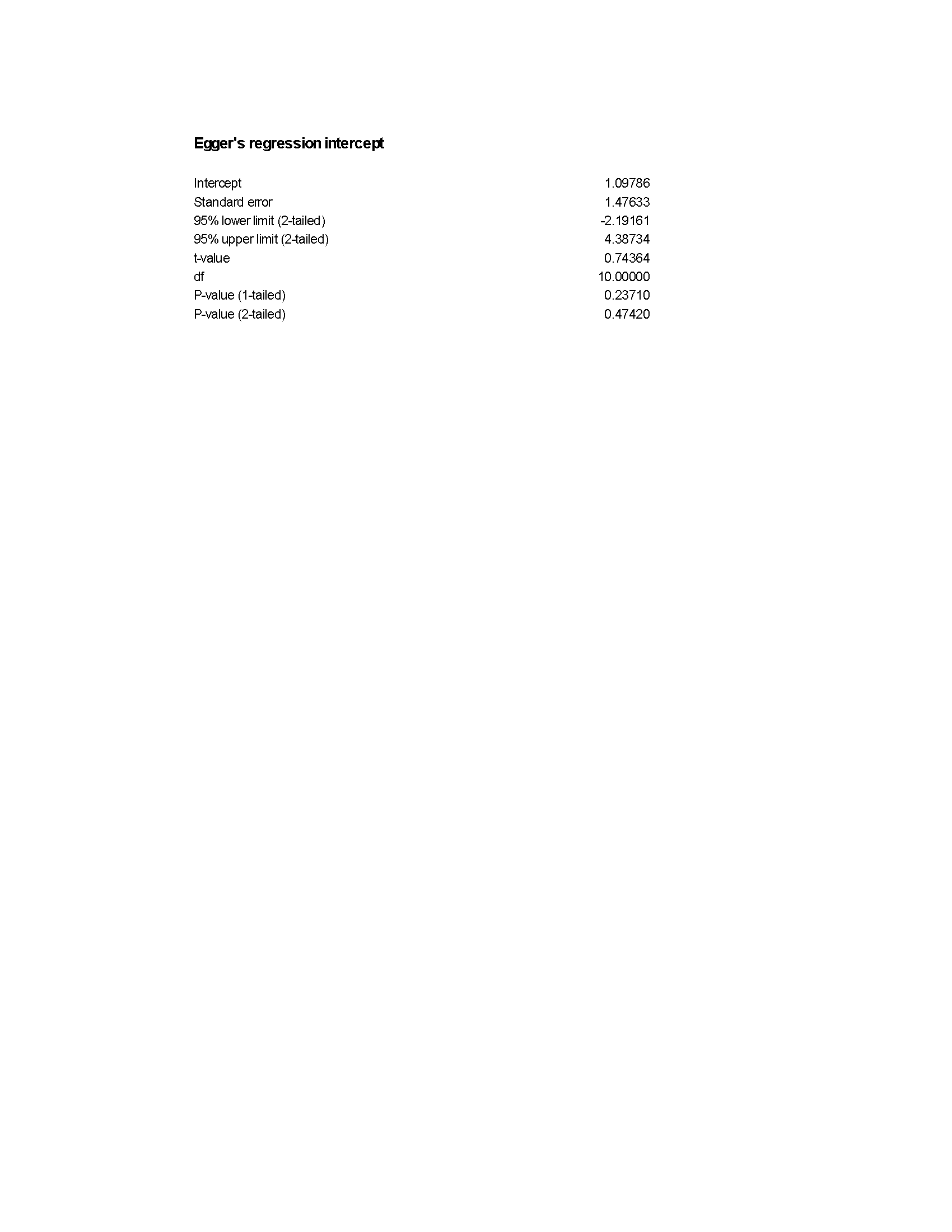

Supplement: Supplementary file 2 [file Table2.DOCX]
